# Exact Stoichiometry Far-Off Neutrality Catanionic Nanotubes: Unconventional Self-Assembly of Oppositely Charged Surfactants

**DOI:** 10.1021/jacs.5c21811

**Published:** 2026-04-30

**Authors:** Valerio La Gambina, Lorenzo A. Rocchi, Simona Sennato, Milad Radiom, Karin Schillén, Crispin Hetherington, Iolanda Francolini, Fabrizio Vetica, Francesca Leonelli, Alessandra Del Giudice, Maria Chiara di Gregorio, Raffaele Mezzenga, Luciano Galantini

**Affiliations:** † Department of Chemistry, 9311 Sapienza University of Rome, Rome 00185, Italy; ‡ Department of Physics, Sapienza University of Rome, Rome 00185, Italy; § National Research Council–Institute for Complex Systems (CNR-ISC), Rome 00185, Italy; ∥ Department of Health Sciences and Technology, ETH Zürich, Zürich 8092, Switzerland; ⊥ Division of Physical Chemistry, Department of Chemistry, Lund University, Lund SE-22100, Sweden; # National Center for High Resolution Electron Microscopy, Centre for Analysis and Synthesis, 101103 Lund University, Lund SE-22100, Sweden; ¶ Department of Materials, 27219 ETH Zurich, Zürich 8093, Switzerland

## Abstract

Electrostatic energy plays a pivotal role in the minimization of the free energy of self-assembling oppositely charged ions. This is a fundamental principle that guides the formation of aggregates and the separation of phases characterized by a stoichiometric balance between the opposite charges. However, this study unveils the self-assembly of catanionic nanotubes from a mixture of oppositely charged surfactants in aqueous buffer, comprising an anionic bile salt derivative and the cationic cetyltrimethylammonium bromide (CTAB) at an exact strongly unbalanced charge ratio of 9 anions to 1 cation. This anomalous aggregation leads to the formation of highly stable and ordered supramolecular nanotubes with a uniform cross-section of about 20 nm. The key to this behavior lies in the unique, rigid, and facial amphiphilic structure of the bile salt derivative, allocating hydrogen bond donors/acceptors and an aromatic residue, which promotes geometrically constrained derivative–derivative interactions. This facilitates the assembly of the anionic derivative into parallel, helically wrapped, negatively charged ribbons with interspersed CTAB cations, acting to promote their adhesion. Supramolecular nanotubes are highly valued in nanotechnology for applications such as catalysis and tissue engineering due to their rigidity, intrinsic directionality, and capability for dual compartmentalization (inner cavity and outer surface). The catanionic structures reported here are ordered architectures that allow for a high charge and a minimal interference from free monomers, which are essential for optimizing functions such as material loading. A loading ability was demonstrated here by the nanotube interaction with positively charged carbon dots and gold nanorods.

## Graphical abstract

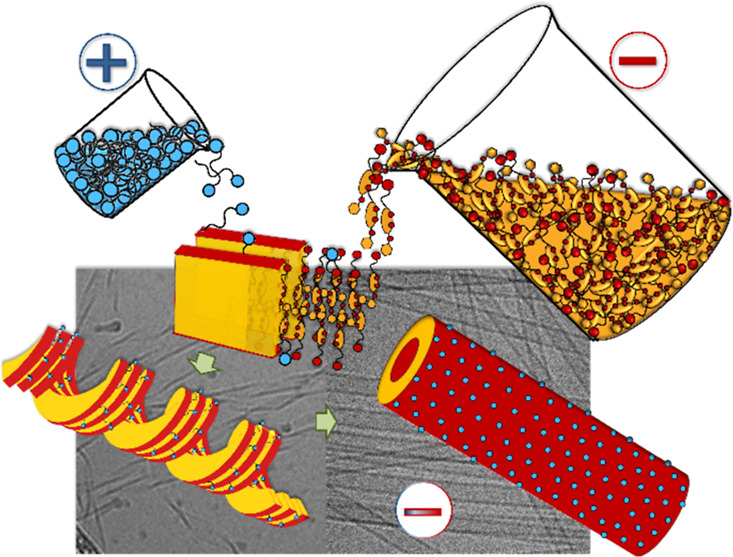

## Introduction

The primary mechanism promoting aggregation within ionic solutions is electrostatic attractive interaction, which typically culminates in the separation of neutral phases.^1,2^ A classic example is the formation of stoichiometric associations at a charge ratio equal to one when saturation is reached in aqueous mixtures of strong inorganic electrolytes, leading to the precipitation of uncharged crystalline structures. Even though hydrophobic contributions promote a more intricate self-assembly mechanism,^3^ the formation of precipitates at equimolar charge composition similarly occurs in mixtures of oppositely charged organic ions. This is frequently demonstrated in mixtures of conventional anionic and cationic surfactants with comparable alkyl chain lengths, where the catanionic surfactant characteristically phase separates at equimolar ratios of the two amphiphiles.^4–11^ Aggregation deviating from this specific composition can occur in these systems, resulting in the formation of stable vesicles characterized by an unbalanced charge ratio.^6–9^ However, this association generally takes place across a wide range of mixture compositions and produces vesicles that lack a single specific stoichiometry. Instead, they feature a distribution of surfactant ratios and sizes. The breadth and the mean of this distribution depend on the mixture’s surfactant.^11–14^ This behavior also holds true in mixtures of surfactants with significantly different molecular structures.^15–17^ Systems with particularly narrow one-phase vesicle regions have been sometimes identified.^18–21^ In addition, thermodynamic models have been developed that predict a sharp distribution of vesicle composition at a fixed surfactant ratio of the mixture.^22^ An association at compositions around the equimolar ratio and no significant aggregation outside is instead observed in mixtures of unconventional steroidal amphiphiles,^23^ without phase separation. Diverging from what is currently observed so far, we unveil here a catanionic surfactant mixture providing a far-off neutrality exact stoichiometry association with the formation of monodisperse and stable tubular aggregates (Figure 1). The mixture comprises the conventional head–tail cationic surfactant cetyltrimethylammonium bromide (CTAB) and the derivative of the anionic bile salt surfactant sodium cholate (ACD) (Figure 1). The nanotubes feature the specific composition of 9 ACD anions per CTAB cation and a highly ordered supramolecular packing. Biological membranes exhibit the interesting capability to shape into nanotubular architectures. This property is observed in diverse biosystems such as bacteria, archaea, viruses, plants, and mammals and is related to a plethora of functions such as material transport, cross-feeding, light harvesting, and signaling.^24–28^ Supramolecular networks of nanotubes and fibrils comprise the cytoskeleton and regulate cellular mechanical properties and intracellular signaling processes.^29^ Inspired by nature, the design and engineering of 1D hollow architectures arising from molecular self-assembly have been an intriguing topic in chemistry.^30^ Although the initial studies on synthetic soft nanotubes date back about 40 years,^31,32^ the synthesis of increasingly sophisticated tubular structures still raises open discussions on fundamental aspects such as the mechanism of formation,^33–38^ transformation,^39^ and interaction with the environment or guest objects.^40,41^ These fundamental aspects turn out to be crucial for enabling an increasingly tailorable design of systems for targeted functions. Indeed, nanotubular structures are a resource for several material and biooriented applications, mainly benefiting from their large surface area, elongated morphology, wide flexibility in terms of surface functionalization, and stimuli responsiveness. For example, self-organized nanotubes have been used as carriers and support for differently sized active materials, exploiting the interior space, wall, and exterior surface as compartments for cargo storage.^42–44^ Other applications have employed nanotubes as connection nanochannels^45,46^ and guiding structures to align and shape nanomaterials.^47,48^ As a general property of catanionic aggregates, catanionic nanotubes feature the additional ability to form at very low concentration of the components, which allows them to withstand dilution and to interact with drugs and other materials without significant interference from free monomers.^49,50^ These properties have been widely proved for the conventional catanionic vesicles and exploited in application like drug delivery^11,51^ and synthesis of novel materials.^52,53^ Nanotubes have been obtained by the assembly of cationic, anionic, nonionic surfactants, and zwitterionic phospholipids.^54^ However, the formation of tubular architectures is a rare event in catanionic systems. Hofmann et al. described in 2008 the uncommon behavior of catanionic mixtures composed of anionic stiff aromatic anions (2-phenylbenzimidazole-5-sulfonic acid) and CTAB.^17^ It was observed that vesicle phases formed in mixtures richest in anionic surfactant after sample preparation and transformed into nanotubes upon aging. Regardless of the mixture composition, it was proposed that a bilayer with an interdigitated CTAB-anion structure with a 1:1 ratio forms the nanotube’s wall core. We previously showed that cationic and anionic derivatives of bile salts can also provide catanionic tubules.^23^ However, in agreement with the conventional behavior of catanionic mixtures, the strongest association was observed at equimolar ratio of the two components in the mixtures.^23^ The formation of catanionic tubules is in this case ascribable to the specific structure of the bile acid derivatives, in which the molecular rigidity and the presence of groups able to form geometrically constrained interactions (e.g., hydrogen bonds and π–π interactions) promote self-assembly into ordered and rigid supramolecular aggregates. We demonstrated here that when mixed with conventional oppositely charged head–tail surfactants, these intermolecular interactions are strong enough to stabilize catanionic nanotubes with a much larger fraction of the bile acid derivative, thereby bending the common tendency to neutrality of catanionic aggregates.

**Figure 1:**
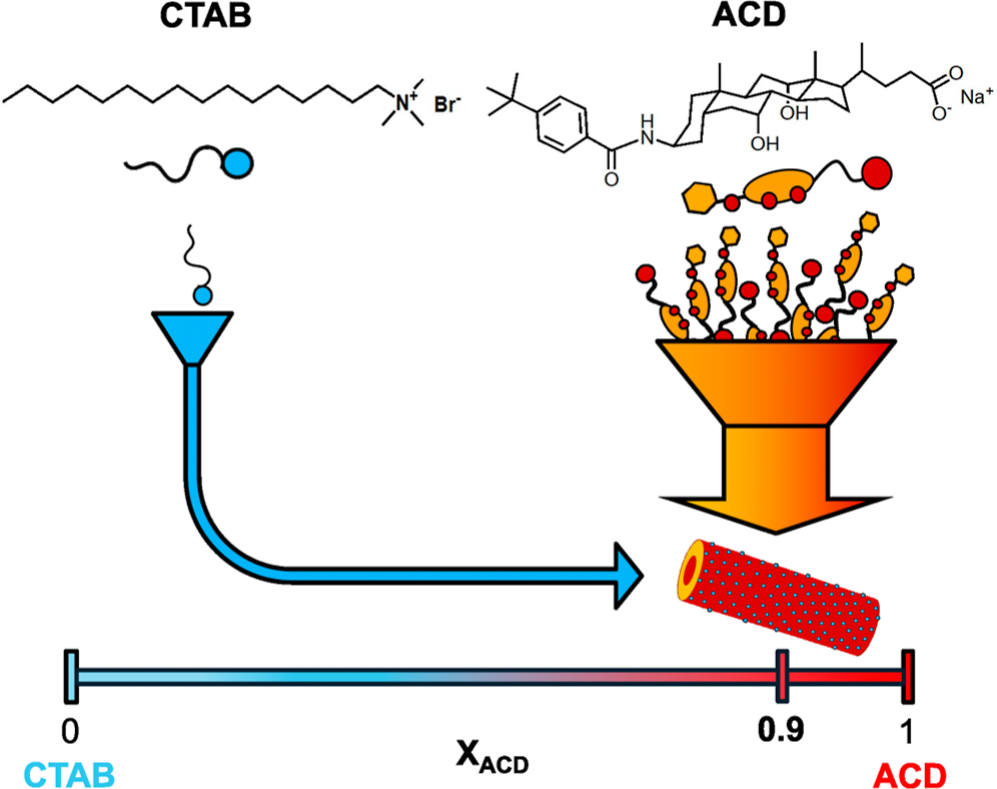
Molecular structures of the cationic surfactant CTAB and the anionic bile salt derivative ACD and their coassembly into monodisperse catanionic nanotubes featuring a molar fraction of ACD (*X*_ACD_) equal to 0.9, that is very far from equimolar composition.

## Experimental Section

### Materials

Sodium bicarbonate (NaHCO_3_, 99.5%), sodium carbonate (Na_2_CO_3_, 99.5%), dimethyl sulfoxide-d6 (DMSO-*d*_6_, 99.5 atom % D), hexadecyltrimethylammonium bromide (CTAB, ≥99%), 1-decanol (*n*-decanol, 98%), hydrogen tetrachloroaurate trihydrate (HAuCl_4_, ≥99.9%), silver nitrate (AgNO_3_, ≥99.0%), l-ascorbic acid (L-AA, ≥99%), sodium borohydride (NaBH_4_, 99%), hydrochloric acid (HCl, 37%), and (11-Mercaptoundecyl)-*N*,*N*,*N*-trimethylammonium bromide (MABr, >95%) were purchased from Sigma-Aldrich and used without further purification. Milli-Q grade water (resistivity 18.2 MΩ·cm at 25 °C) was used in all experiments. The synthesis of ACD was previously reported.^23^

### Preparation of ACD-CTAB Catanionic Mixtures

The self-assembly of the catanionic mixtures was studied on samples with a total concentration of surfactants ranging from 0.6 to 10 mM in NaHCO_3_/Na_2_CO_3_ buffer solutions (pH = 10). The lowest investigated total surfactant concentration was close to or below the c.m.c of the pure components, which have been reported to be about 0.5 mM for ACD in 30 mM NaHCO_3_/Na_2_CO_3_ buffer^55^ and 0.9 mM for CTAB in water.^56^ The effect of the buffer concentration on self-assembly was also investigated by varying the concentration from 15 to 90 mM. Catanionic mixtures were prepared by mixing appropriate volumes of stock solutions of ACD and CTAB in NaHCO_3_/Na_2_CO_3_ buffer. Both stock solutions of ACD and CTAB were prepared by dissolving their respective powders in NaHCO_3_/Na_2_CO_3_ buffer, followed by vortexing for about 2 min. Stock solutions in the 0.3–10.0 and 1.0–10.0 mM ranges were used for CTAB and ACD, respectively. After preparation, the ACD-CTAB mixtures required an equilibration period to allow aggregate formation, which was dependent on the concentration of the buffer. The mixture’s composition was reported as the ACD surfactant molar fraction defined as *X*_ACD_ = *n*_ACD_/(*n*_ACD_ + *n*_CTAB_), where *n*_ACD_ and *n*_CTAB_ are the number of moles of ACD and CTAB, respectively. Mixtures with *X*_ACD_ values from 0.975 to 0.3 were investigated.

### Synthesis of Carbon Dots

Carbon dots were synthesized according to the procedure previously described.^57^ Briefly, 1,3-diaminobenzene (550.0 mg, 5.09 mmol) was completely dissolved in MeOH by sonication (10 min) before mixing it with d-glucosamine hydrochloride (1.0 g, 4.64 mmol) (d-glucosamine hydrochloride:1,3-diaminobenzene ratio = 1:1.1) and water to reach a final solution of water/MeOH (2:1, 30 mL total). The resulting solution was then carbonized in a microwave at 800 W for 3 min. The resulting slurry solution was diluted in Milli-Q water (10 mL) and centrifuged at 4000 rpm to remove larger particles. The supernatant was directly dialyzed for 3 days (the dialysis water was changed at least 5 times per day) and then filtered through a 100 nm syringe filter. The resulting solution was freeze-dried to obtain the desired carbon dots.

### Preparation of the Catanionic Mixture-Carbon Dots Hybrids

Catanionic mixtures at *X*_ACD_ = 0.9 in 30 mM NaHCO_3_/Na_2_CO_3_ buffer, forming stable nanotubes, were selected for the interaction with positively charged carbon dots. In a typical experiment, a stock solution of carbon dots (0.5 mg/mL), prepared by dissolving the carbon dots in Milli-Q water, was mixed with a 1.0 mM stock solution of the catanionic mixture at a 3:1 catanionic/carbon dot solution volume ratio.

### Synthesis of MABr Stabilized Gold Nanorods

Gold nanorods were synthesized according to the procedure previously described.^58^ Briefly, a solution containing 50 mM CTAB and 13.5 mM *n*-decanol was prepared by dissolving 9.111 g of CTAB and 1.068 g of *n*-decanol in 500 mL of Milli-Q water. The mixture was stirred at 35 °C for 3 h until complete dissolution.

Synthesis of 1–2 nm Gold Seeds: after complete dissolution of CTAB, 20 mL of the CTAB/n-decanol solution was transferred into a beaker and equilibrated at 25 °C. Then, 200 μL of 50 mM HAuCl_4_ and 100 μL of 0.1 M L-AA were added. The solution was stirred until it became completely transparent. Once transparency was achieved, 800 μL of 20 mM NaBH_4_ was rapidly injected under vigorous stirring, resulting in a brown–yellow coloration. The solution was left undisturbed for 1 h prior to use in order to allow decomposition of excess sodium borohydride.

Synthesis of Gold Nanorods: 300 mL of the 50 mM CTAB/13.5 mM decanol solution was transferred into an Erlenmeyer flask at 25 °C, followed by the addition of 2.4 mL of 10 mM AgNO_3_, 21 mL of 1 M HCl, and 3 mL of 50 mM HAuCl_4_. Subsequently, 3.9 mL of 0.1 M L-AA was added, and the mixture was stirred until it became completely transparent. Finally, 18 mL of the 1–2 nm seed solution was added under stirring. The reaction mixture was then left undisturbed for 4 h. After 4 h, the obtained solution was centrifuged at 14,800 rpm for 1 h, the supernatant was discarded, and the particles were redispersed in 30 mL of 0.5 mM MABr. The solution was allowed to rest for 1 h to enable MABr interaction with the nanorods. Subsequently, the solution was recentrifuged at 14,800 rpm for 1 h and redispersed in Milli-Q water. This procedure was repeated three times to remove excess CTAB. Finally, the [Au^0^] concentration was adjusted to 3 mM by monitoring the absorbance at 400 nm.

### Preparation of the Catanionic Mixture-Gold Nanorods Hybrids

Catanionic mixtures at *X*_ACD_ = 0.9 in 30 mM NaHCO_3_/Na_2_CO_3_ buffer, forming stable nanotubes, were selected for the interaction with positively charged gold nanorods. In a typical experiment, a solution of gold nanorods ([Au^0^] = 3 mM) was mixed with a 1.0 mM stock solution of the catanionic mixture at a 3:1 catanionic/gold nanorods solution volume ratio.

### UV Absorption and Circular Dichroism

UV absorption (UV) and circular dichroism (CD) measurements were performed by using a Jasco J-715 polarimeter equipped with a xenon lamp and a Peltier unit for temperature control. Spectra were collected over the 190–450 nm wavelength range. Quartz cuvettes with a path length of 0.1 mm were used. All of the reported spectra are the results of 4 scans. Each scan was collected at an accumulation speed of 100 nm/min and a resolution of 0.2 nm. Both the CD and UV spectra were corrected for the buffer contribution and reported as molar ellipticity θ and molar extinction coefficient ε, respectively. A range of temperature between 20 and 55 °C has been investigated. After each temperature change, the sample was left to stand for 10 min, and *g*-factors were calculated as

$$g‐factor=\frac{CD/mdeg}{32980^{*}Extinction}$$

### Small Angle X-ray Scattering

Small angle X-ray scattering (SAXS) measurements were performed at SAXSLab Sapienza with a Xeuss 2.0 Q-Xoom system, equipped with a microfocus Genix 3D X-ray source at wavelength λ = 0.1542 nm and a two-dimensional Pilatus3 R 300 K detector (Dectris Ltd., Baden, Switzerland). Calibration of the scattering vector q range, where *q* = 4π sin θ/λ and 2θ is the scattering angle, was performed using silver behenate. Samples were loaded into vacuum tight quartz capillary cells with a thickness of 1.5 mm and measured in the instrument sample chamber at reduced pressure (0.2 mbar) in a thermalized holder at 21 °C. Measurements were performed for a total exposure time of 8 h per sample with three different sample–detector distances so that the overall explored q region was 0.045 nm^–1^ < *q* < 17 nm^–1^. The two-dimensional scattering patterns were subtracted for the “dark” counts, and then masked, azimuthally averaged, and normalized for transmitted beam intensity, exposure time, and subtended solid angle per pixel, by using the FoxTrot software developed at SOLEIL. The intensity vs q profiles were then subtracted for the background contribution of the Na_2_CO_3_/NaHCO_3_ buffer solvent measured in the same cell and put in absolute scale units (cm^–1^) by dividing by the capillary thickness obtained by calibration with water. The different angular ranges were merged using the SAXS utilities tool.^59^ Additional SAXS measurements were performed at the BM29 bioSAXS beamline (ESRF, Grenoble)^60^ with X-ray wavelength λ = 0.0992 nm and sample-to-detector distance 281 cm, allowing data collection in the scattering vector range 0.07 nm^–1^ < *q* < 5 nm^–1^. Samples were measured at 20 °C using the automatic sample changer mode of acquisition, alternating sample, and buffer measurements. Ten subsequent 1 s exposures were collected while flowing 50 μL of sample in the 1.0 mm quartz capillary, and the 2D images were masked, azimuthally averaged, and converted to absolute unit scale (using water as a standard) by the automatic pipeline. When the partial alignment under flow of the elongated aggregates resulted in anisotropic scattering patterns, the automatically averaged data over the full azimuthal angle showed a small distortion of the profile in correspondence with secondary maxima. To remove this artifact due to preferential orientation, image integration under a limited sector of 15° in the direction perpendicular to the flow was applied, and the result was scaled to the intensity of the fully azimuthally averaged data. Subsequent frames were checked for overlap, averaged, and subtracted for the background contribution measured for the buffer. Model fitting was performed with SasView software.

### Atomic Force Microscopy

Atomic force microscopy (AFM) measurements have been performed by depositing a 5 μL drop of each sample on a freshly cleaved mica support (Ted Pella) functionalized with (3-aminopropyl)­triethoxysilane (APTES, Sigma). Both rinsing with Milli-Q water after 3 min of incubation and simple evaporation under ambient conditions were considered in the sample preparation. Measurements were performed by a Bruker multimode 8 scanning probe microscope (Bruker, U.S.A.) with an acoustic hood to minimize vibrational noise using RTESPA-150 (Bruker) probes (nominal radius of curvature 8 nm) and by a Dimension Icon (Bruker AXS) using RTESP-300 (Bruker) probes (nominal radius of curvature 10 nm). Samples were analyzed under ambient conditions in Soft Tapping Mode. Background subtraction on height sensor channel data was performed by line-by-line or three-point data leveling using Nanoscope 8.1. Flattened images were processed by using Gwyddion.

### Dynamic Light Scattering and Electrophoretic Mobility

Dynamic light scattering (DLS) and electrophoretic mobility measurements were performed by using a Malvern NanoZetaSizer, equipped with a 5 mW He–Ne laser (λ = 632.8 nm) and a digital logarithmic correlator. The normalized intensity autocorrelation functions were measured at a scattering angle of 173° at 25.0 °C. For DLS measurements, every sample was analyzed for 1 min, and a UV-microcuvette of volume 70 μL was used. The General Purpose analysis mode based on the non-negative least-squares algorithm was used to extract the intensity-weighted relaxation time distributions. Monomodal distributions were obtained for all of the samples. In the case of electrophoretic mobility measurements, 15 runs for each sample were collected at the scattering angle of 13° using a disposable folded capillary cell filled with 1 mL of sample solution.

### Transmission Electron Microscopy

Cryogenic transmission electron microscopy (cryo-TEM) images were collected on a TVIPS F416 camera using a 200 kV JEM-2200FS in Lund. The samples were prepared by using a Leica GP1 controlled environment vitrification system. A drop of the sample was deposited on a Cu 200 mesh TEM grid, coated with a lacey Formvar/carbon holey film (Ted Pella Ltd., part 01881). The grid was blotted to remove excess fluid, resulting in a thin film (20–300 nm) of the solution suspended over the grid’s holes. The samples were vitrified by rapid plunging into liquid ethane at −184 °C, transferred to a cryo transfer tomography holder (Fischione model 2550) and examined at around −175 °C. TEM images were collected using a JEOL JEM F200 (equipped with a CMOS Camera System) operating at 80 kV. The samples were prepared by depositing 5 μL of the mixture on a Cu TEM grid coated with a Formvar/carbon film (300 mesh, Ted Pella, Ltd.). It was left for 10 min to allow the complete grid permeation, and after that, the grid was deposited on filter paper for absorbing the drop excess.

### Scanning Electron Microscopy

Scanning electron microscopy measurements (SEM) were performed with a HR-FESEM AURIGA ZEISS instrument, operating between 3 and 5 kV voltage. InLens or mixed secondary electron-InLens imaging was used by means of an Everhart Thornley Detector. Samples were prepared by dropping 5 μL of the solution on silicon plates and letting it dry overnight.

### Attenuated Total Reflectance Fourier-Transform Infrared Spectroscopy

Attenuated total reflectance Fourier-transform infrared spectroscopy (ATR-FTIR) spectra were acquired by using a Bruker Lumos II micro-FTIR ATR spectrometer equipped with a Ge crystal. Spectra were recorded in the 4000–550 cm^–1^ spectral range with a resolution of 4 cm^–1^ by averaging 1024 scans. Background spectra were collected against air prior to each measurement. No baseline correction was carried out. All measurements were performed at room temperature. Specimens were prepared by depositing approximately 40 μL of the sample solution onto silicon wafers. The deposition step was repeated three times, followed by solvent evaporation overnight. The resulting dried samples were directly analyzed in the ATR mode.

### Proton Nuclear Magnetic Resonance

Proton nuclear magnetic resonance (^1^H NMR) analyses were performed on a 400 MHz NMR Bruker Avance NEO 400 Nanobay spectrometer. Individual samples of ACD and CTAB (6.0 mg/mL) were dissolved in DMSO-*d*_6_ at room temperature and analyzed using the residual solvent signal as an internal standard. Spectra were processed using MestReNova 6.0.2 (Mestrelab Research SL). The catanionic mixture at *X*_ACD_ = 0.8 was analyzed following two preparation protocols. In the first protocol, the mixture solution was dried under a vacuum without prior separation, and the resulting solid residue was redissolved in DMSO-*d*_6_ before NMR acquisition. In the second protocol, a 0.5 mL aliquot of the *X*_ACD_ = 0.8 mixture was centrifuged at 14 800 rpm for 1 h. The resulting pellet, containing the separated aggregates, and the corresponding supernatant were isolated and dried under a vacuum. The resulting solids from both fractions were redissolved in DMSO-*d*_6_ and analyzed, collecting 1024 scans for each spectrum.

## Results and Discussion

### Catanionic Aggregates’ Morphology and Composition

A 1 mM ACD solution in 30 mM carbonate/bicarbonate buffer self-assembles into fibrillar structures, as evidenced by both Cryo-TEM and AFM images (Figure 2a). The Cryo-TEM inferred cross-section and the AFM height profile are about 5 and 4 nm, respectively (Figure S1c). At the same total surfactant concentration, catanionic mixtures of ACD and CTAB in 30 mM carbonate/bicarbonate buffer exhibit a completely different assembly. The DLS measurements distinctly revealed a significant change in the diffusive properties for the mixture around *X*_ACD_ = 0.9, showing a sharp increase in the relaxation time due to aggregation. At the same time, there was an increase in the total light scattering intensity at this composition (Figure 2b).

**Figure 2:**
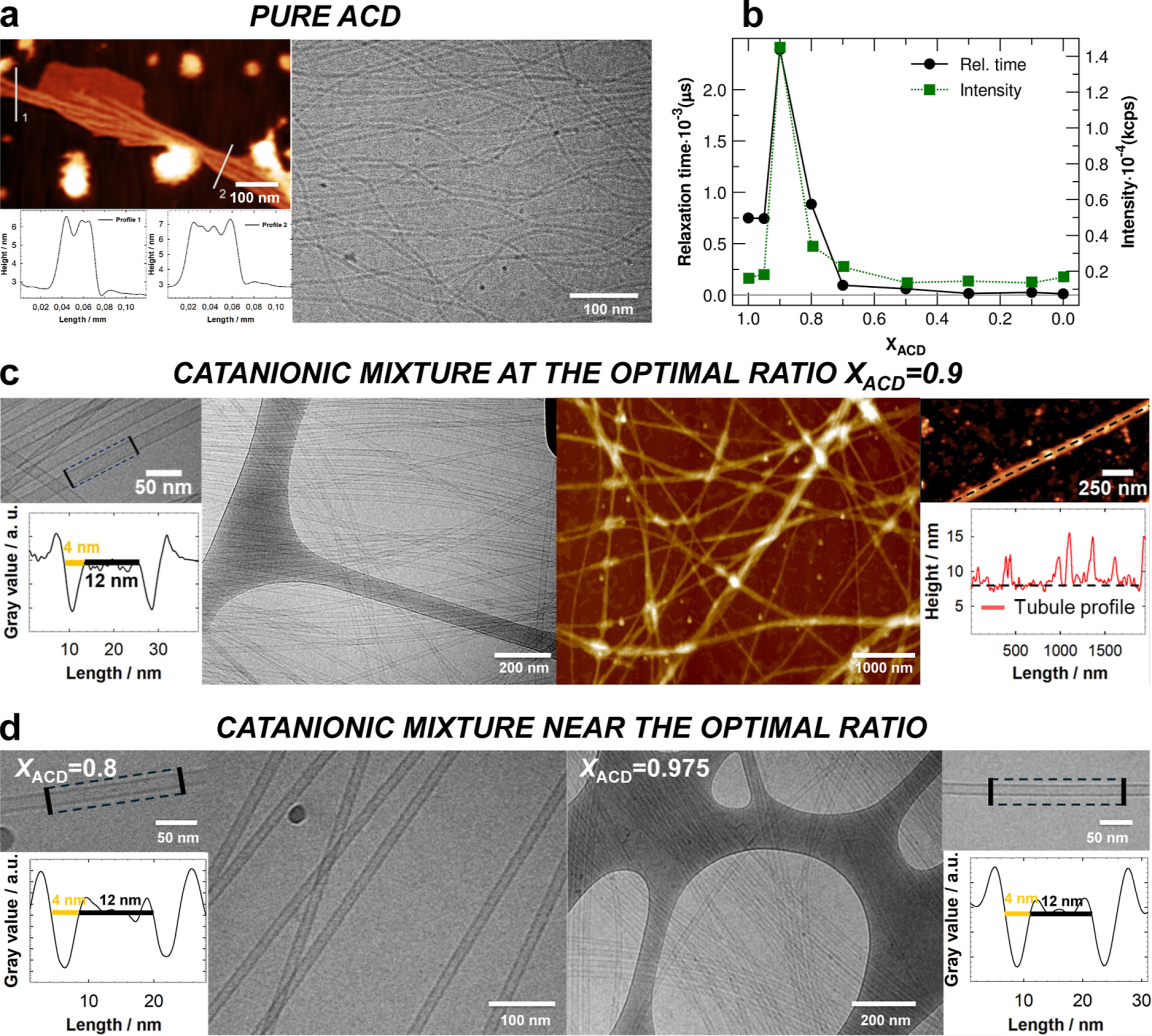
(a) AFM and Cryo-TEM images of ACD (1 mM) fibers in 30 mM carbonate/bicarbonate buffer. (b) Relaxation time from DLS measurements and average total light scattering intensity as a function of the *X*_ACD_ for mixtures at a total surfactant concentration of 1 mM. (c) Cryo-TEM and AFM images of catanionic tubes formed at *X*_ACD_ = 0.9, at a total surfactant concentration of 1 mM in 30 mM carbonate/bicarbonate buffer. (d) Cryo-TEM images of catanionic tubes formed at *X*_ACD_ values close to the optimal ratio (*X*_ACD_ = 0.8, left; *X*_ACD_ = 0.975, right) at a total surfactant concentration of 1 mM in 30 mM carbonate/bicarbonate buffer.

Cryo-TEM imaging of the sample at *X*_ACD_ = 0.9 revealed well-defined nanotubes with an average diameter of 20 ± 1 nm and a wall thickness of 4 ± 1 nm (Figures 2c and S1a,b). Interestingly, such a wall thickness is very similar to the ACD fibrils cross-section diameter. The nanotubes extend over several microns in length and were also detected at nearby compositions within the range *X*_ACD_ = 0.8–0.975, without any detectable change in their cross-sectional dimensions and wall thickness (Figures 2d and S2a,b). The nanotubes were also preserved after drying and could be imaged by AFM. AFM images of the *X*_ACD_ = 0.9 sample (Figures 2c and S3) showed that the profile of clean nanotubes exhibited heights of about 8 nm, which is consistent with a wall thickness of about 4–5 nm, considering the flattening of nanotubes on the APTES-mica support.

Consistently with DLS data, the SAXS curves collected in the *X*_ACD_ range of 0.7–1.0 showed the most intense scattering signal at *X*_ACD_ = 0.9 (Figure 3a,c). A well-defined oscillating pattern with an initial q^–1.7^ slope was recorded at this composition. These features are typical of long tubular scattering particles, and the curve could be well reproduced by using the form factor of a long hollow cylinder with a cross-section inner diameter of 11.5 ± 0.5 nm and wall thickness of 4.0 ± 0.1 nm (Figure 3b). Although the characteristic power law of the lowest q intensity decay was approximately maintained, the oscillations gradually disappeared in the SAXS curves at both higher and lower *X*_ACD_ values, in parallel with the lowering of the intensity (Figure 3c). A diagram of the hollow cylinder volume fraction as a function of composition (Figure 3c, inset) further underscores *X*_ACD_ = 0.9 as the condition yielding the most efficient nanotube formation.

**Figure 3:**
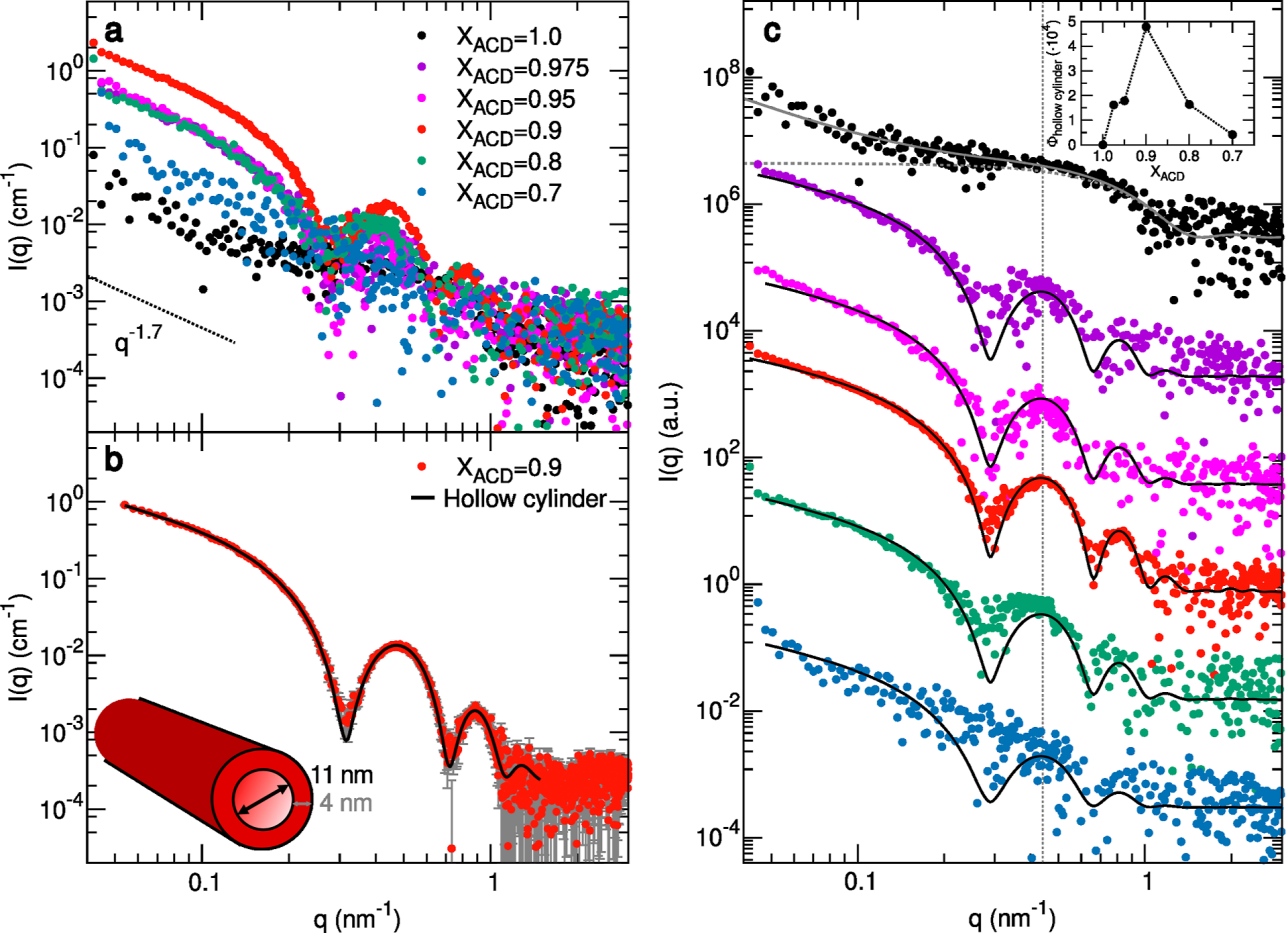
(a) SAXS curves of 1 mM pure ACD solution and the ACD/CTAB mixtures in 30 mM carbonate/bicarbonate buffer at different ACD fractions, reported as macroscopic scattering cross-sections on an absolute scale. (b) Higher signal-to-noise ratio SAXS data of the *X*_ACD_ = 0.9 mixture fitted with the form factor of a long hollow cylinder with an overall diameter of 19 nm and a wall thickness of 4 nm. (c) SAXS data of panel (a) are shown shifted by a suitable factor for improved visualization, together with model form factors of a long hollow cylinder for the mixtures with 0.7≤ *X*_ACD_ ≤ 0.975 and, for the pure ACD solution, of spherical micelles with a small contribution of flexible cylinders. In the inset, the hollow cylinder’s volume fraction is reported as a function of the ACD fraction. Best fitting parameters are reported in Tables S1, S2, and S3.

The remarkably uniform distribution of tube diameter and wall thickness across the *X*_ACD_ = 0.8–0.975 range indicates that the nanotubes adopt a well-defined stoichiometry that is maintained independently of the overall mixture composition. To verify this conclusion, the nanotubes were separated upon centrifugation from the mixture at *X*_ACD_ = 0.8. They were then solubilized in deuterated DMSO and analyzed by ^1^H NMR. A *X*_*ACD*_ = 0.87 was inferred for the nanotubes by comparing the integrated peaks of the *N*-methyl protons of CTAB at 3.02 ppm [s, 9H, N^+^(CH_3_)_3_] and the aromatic protons of ACD at 7.45 ppm [m, 2H, C_Ar_-H] and 7.70 ppm [m, 2H, C_Ar_-H] (Figure S4). Considering that a small fraction of the CTA anions can adsorb to the nanotube pellet upon separation, the obtained *X*_ACD_ fraction is consistent with a composition of 9 ACD anions per CTA cation.

### Molecular Packing Characterization

The catanionic mixtures showed a characteristic UV spectrum with absorption bands around 200 and 240 nm that are associated with electronic transitions of the phenylamide group of ACD (Figure 4a). In the same range of absorption wavelengths, CD signals were observed for the mixtures at the highest *X*_ACD_ values (*X*_ACD_ ≥ 0.8) at 30 mM carbonate/bicarbonate buffer (Figure 4b,c). The CD profiles were overall invariant with respect to composition and drastically different from those of pure ACD, showing a negative peak at the lowest recorded wavelength (190 nm), two positive bands at 207 and 220 nm, and a negative band centered at 246 nm. The observed CD response indicates that the self-assembly of the surfactants occurs in these mixtures, which implies a relevant interaction and chiral packing of the transition dipoles associated with the aromatic residues of the ACD molecules. The chiral packing of the chromophores differed from that occurring in pure ACD solution, for which a conservative bisignate Cotton effect consistent with a left-handed helical arrangement of their transition dipoles was detected (Figure 4c). The CD signal intensity depends on the mixture composition. The diagram of the molar ellipticity at 240 nm shows the maximum intensity at *X*_ACD_ = 0.9, where the best conditions for coassembly occur (Figure 4b, inset). Interestingly, the UV spectra parallel the CD curves, showing the highest extinction coefficients at *X*_ACD_ = 0.9, as highlighted in the inset of Figure 4a, where the value of the longer wavelength band is presented as a function of composition.

**Figure 4:**
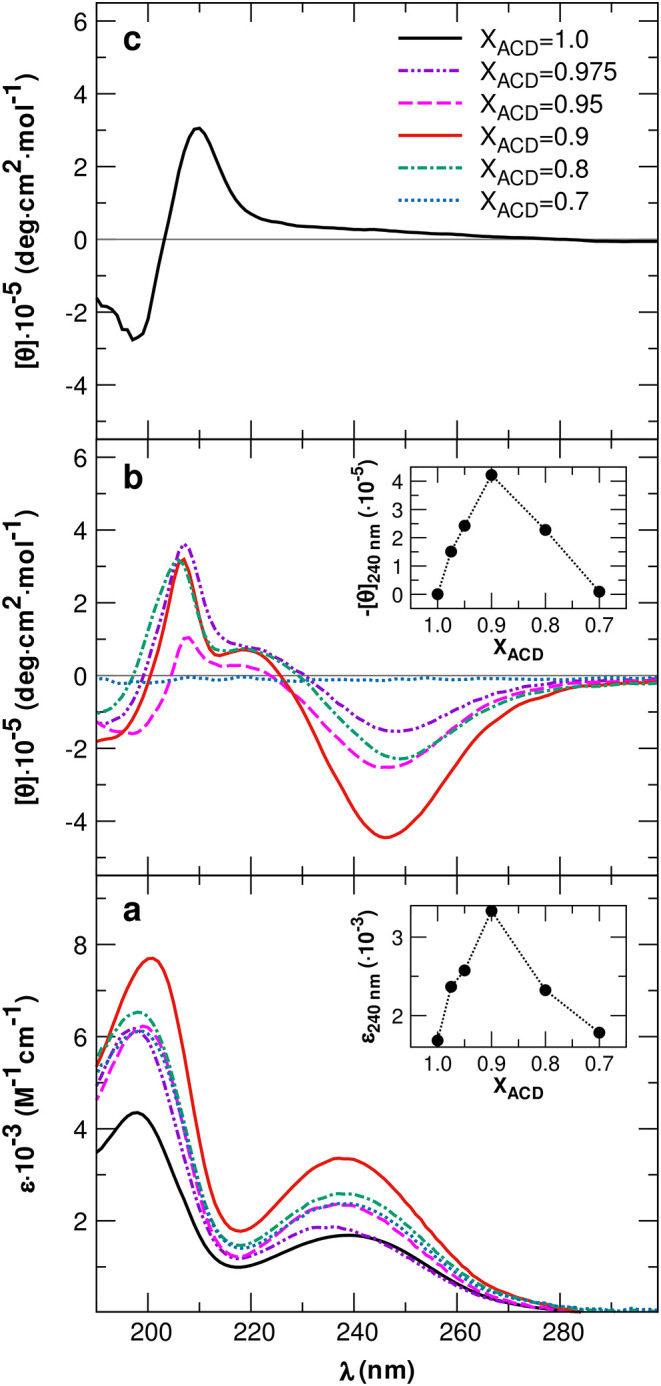
(a) UV absorption curves of the pure ACD solution and the ACD/CTAB mixtures. (b) CD curves of the mixtures at different *X*_ACD_ and (c) of the pure ACD. In the insets, the signal at 240 nm is shown as a function of the ACD molar fraction in the mixture. Total surfactant concentration is 1.0 mM.

A similar behavior was also observed after normalization of the absorbance for the ACD concentration, clearly demonstrating that an enhancement of the absorbance is promoted by self-assembly. Therefore, all of the data point to *X*_ACD_ = 0.9 as the optimal composition for the most efficient self-assembly of the catanionic mixture. Such behavior was also confirmed at a lower total surfactant concentration of 0.6 mM, which is close to the critical aggregation concentration of ACD and where pure ACD shows a negligible self-assembly (Figure S5). It is important to note that the CD intensities scale with the fractions of CTAB at *X*_ACD_ ≥ 0.9. This suggests that 0.9 is also the fraction of ACD in the aggregates and that, with maintaining this fraction, the nanotubes form until complete consumption of CTA cations in this mixture composition range.

The molecular structure of bile acids enables self-assembly by both hydrophobic interactions and hydrogen bonds involving the hydroxyl groups. The comparison of micro-FTIR data collected on dry samples of ACD fibers and ACD/CTAB nanotubes with those obtained by drop casting from a monomeric solution of ACD in methanol clearly demonstrates that the formation of hydrogen bonds is also fundamental in the ACD aggregates (Figure S6). As a matter of fact, the results showed that a significant shift of the OH stretching toward lower wavenumbers (from 3450 to 3250 cm^–1^) occurs by moving from the amorphous methanol solution drop cast sample, representative of the monomeric dispersion, to those of the aggregates. As shown by UV and CD curves, additional interactions of the aromatic residues contribute to the aggregation of the investigated derivative in fiber or mixed nanotubes. FTIR spectra indicate that a slight shift to higher wavenumbers of the amidic carbonyl group stretching occurs because of these interactions, as observed by comparing the signal of the amorphous deposition (1640 cm^–1^) with those of fiber (1650 cm^–1^) or nanotube aggregates (1660 cm^–1^). This is probably due to a slight distortion of the planar structure of the aromatic amide, caused by the intermolecular interactions, which lowers the electronic delocalization, thereby increasing the double bond character of the carbonyl group. The larger stretching wavenumber observed for the mixture sample (1660 cm^–1^) suggests that this effect is particularly strong in the case of the nanotubes, in agreement with the particularly intense UV absorption and CD signals observed for this sample. The carboxylic groups located on the surfaces of the fibers or nanotube walls are expected to form hydrogen bonds with water in solution but are unable to form intermolecular hydrogen bonds since they are far from the hydroxyl and amide groups. These groups are, therefore, free in the dried samples analyzed. Intermolecular hydrogen bonds are instead possible for the carboxylic group in the amorphous methanol drop cast samples, thereby explaining its lower frequency when compared with those of the fiber and nanotube samples. The complex set of interactions, along with the intrinsic chirality of the molecules, makes the self-assembly of these systems very similar to that proposed for peptides,^61^ peptide amphiphiles,^62^ amyloid fibrils,^63–65^ and nematic elastomer tapes.^66,67^ Indeed, previous data reported in the literature demonstrate that bile acid derivatives and peptides share the ability to self-assemble into similar aggregates such as fibrils, twisted and helical ribbons, and nanotubes.^68,69^

### Buffer and Total Surfactant Concentration Effects

We observed that the carbonate/bicarbonate buffer is essential for the self-assembly of the mixture. In fact, DLS measurements of catanionic mixtures in Milli-Q water (Figure S7) showed a maximum at the equimolar ratio and negligible aggregation at other compositions, indicating completely different behavior in the absence of buffer. It is well-known that added electrolyte concentration remarkably affects the phase behavior of catanionic surfactant mixtures.^18^ Accordingly, the self-assembly of our samples pointed out a significant dependence on the buffer concentration. In particular, SAXS profiles reveal that the characteristic oscillatory signature of nanotubes persists at buffer concentrations ≥30 mM and shifts toward lower q values with increasing buffer content, indicating a cross-sectional expansion (Figure S8). This effect is observed up to 60 mM, where an increase in tubule diameter of about 3 nm is indicated by the SAXS curve fit (Figures S8b,c). CD spectroscopy further supports the key role of the buffer (Figure S9). For the *X*_ACD_ = 0.9 sample, a minimum buffer concentration of >15 mM is required to promote nanotube formation. Well-defined nanotubes are observed at 30 mM, where the CD signal reaches its maximum intensity. Upon further increasing the buffer concentration, the CD band intensities gradually decrease until a plateau is reached at ≥ 60 mM. This behavior is consistent with a partial distortion of the supramolecular organization, likely associated with the cross-sectional expansion evidenced by SAXS.

Nanotube structures are preserved at a total surfactant concentration of 2.0 mM, as confirmed by the invariance of both CD and SAXS profiles (Figure S10). Upon increasing the concentration to 10 mM, DLS and SAXS data again indicate that aggregation is most favored at *X*_ACD_ = 0.9 (Figure S11); however, no CD signal is detected, suggesting the absence of well-defined nanotubes under these conditions. Temperature-dependent CD measurements show a progressive decrease in signal intensity with increasing temperature, consistent with gradual nanotube disassembly, with complete loss of signal at T ≥ 48 °C (Figure S12).

### Self-Assembly Model

Time-resolved UV spectra and Cryo-TEM images were collected for mixtures at *X*_ACD_ = 0.9 to infer mechanistic information. The results demonstrated that the UV signal progressively increased over time until reaching a plateau, with a rate that significantly depended on the buffer concentration (Figure S13). The process took 2 h at a buffer concentration of 60 mM or several days at a lower concentration (30 mM). At time zero, no CD signal was detected, suggesting the formation of achiral structures, most likely micellar aggregates, also supported by cryo-TEM (Figure S14). Images at intermediate times after preparation (1 day) showed the presence of nanoribbons (Figure 5b) with a width in the range between 15 and 35 nm and an average value of 25 ± 5 nm (Figure S15a). Helical architecture was observed for all of the ribbons except for the few very thin ones that presented a twisted structure. The cross-section diameters of the helical ribbons ranged from 18 to 37 nm, with an average value of about 25 ± 3 nm (Figure S15b). They progressively closed into final nanotubes with time. An increase in the ribbon width is expected to occur in this closure process. Some details of the micrographs highlight the merging of helical ribbons as a possible mechanism for nanotube formation. The comparison between the average cross-sectional diameter of the helical ribbons and that of the nanotubes (20 nm) suggests that a decrease in diameter occurs in this final step. Reasonably, a tightening of the intermolecular interactions occurs in parallel with the closure of the helical ribbons into nanotubes, which accounts for the final increase in the UV signal.

**Figure 5:**
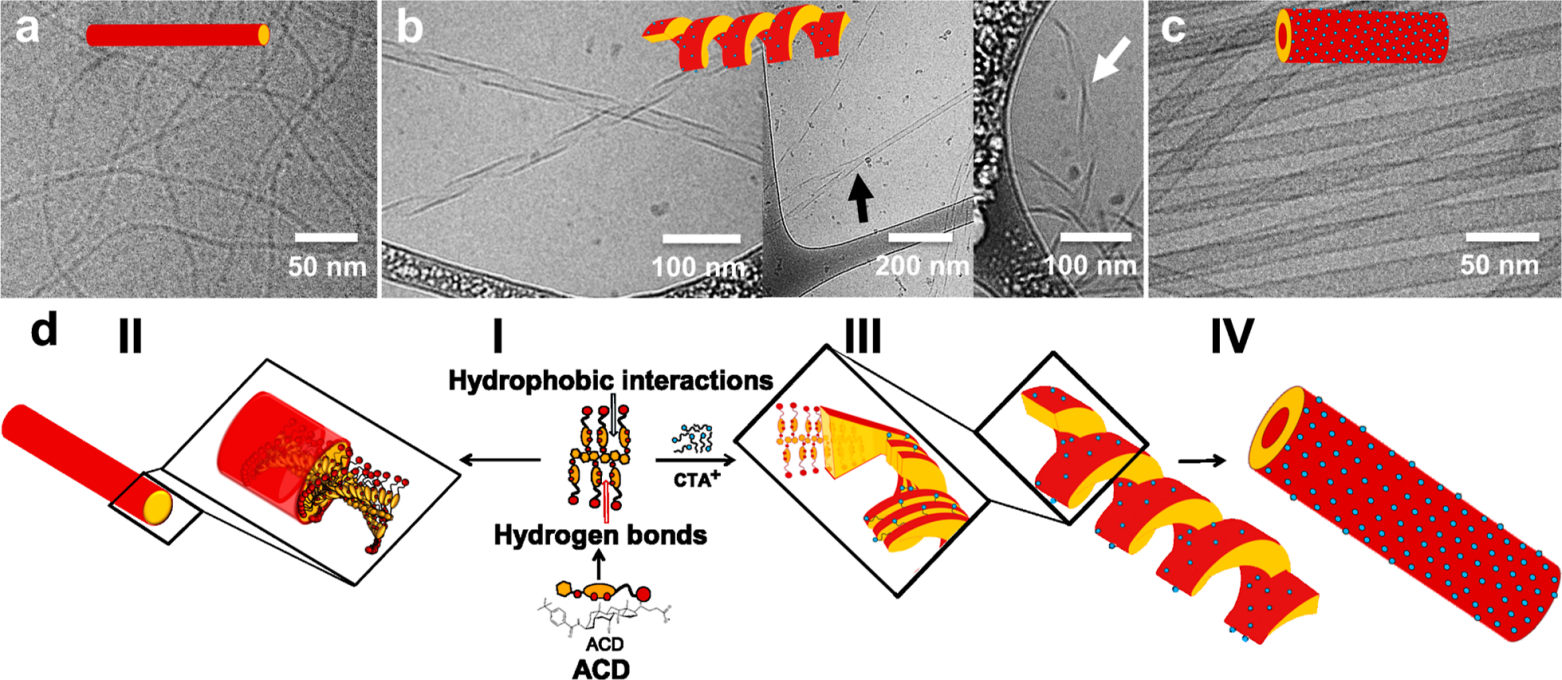
(a) Cryo-TEM image of 1 mM ACD in carbonate/bicarbonate buffer. (b) Cryo-TEM images of the mixture at *X*_ACD_ = 0.9 after 1 day from preparation, showing the presence of helical ribbons, helical ribbons merging (black arrow), and a twisted ribbon (white arrow). (c) Cryo-TEM image of the sample with *X*_ACD_ = 0.9 after 2 weeks from preparation, showing the presence of nanotubes. (d) Proposed model for the formation of the catanionic nanotubes: (I) association of dimers into ribbons, (II) twisted fibrils formed by the pure ACD, (III) thin helical ribbons aligned in parallel due to the presence of CTAB forming a thick ribbon, and (IV) final nanotube.

The reported results allow us to propose models for the aggregate structures of pure ACD solutions and ACD/CTAB mixtures, as shown in Figure 5d.

We consider a dimer as a fundamental unit of these aggregates. The dimer involves a strong interaction between the aromatic groups, in agreement with the UV and CD results. It also implies a distance of about 4 nm between the carboxylic groups, which are expected to be in contact with the medium, thereby providing the local thickness of the ACD/CTAB nanotube walls and roughly of the ACD fibrils, in agreement with the microscopy and SAXS results. In the formation of these structures, an association of the dimers occurs thanks to their ability to form hydrophobic interactions with the apolar faces, hydrogen bonds with the hydroxyl and amide groups, and stacking of the aromatic residues (Figure 5d,I). Due to the chirality of the ACD molecules, adjacent dimers are twisted. The twist is particularly relevant in the aggregation of pure ACD samples and leads to the formation of the elongated fibrils observed in the cryo-TEM images of the same samples (Figure 5d,II). In the formation of the nanotubes, the interaction among ACD molecules is accomplished in ribbons of dimers. The presence of CTAB promotes the adhesion of the ribbons into thicker ones, comprising a bilayer of ACD molecules, by exploiting its ability to screen the carboxylic heads and act as hydrophobic linkers between these molecules. The twist between adjacent ACD molecules imposed by their chirality determines the helical architecture of the bilayer ribbons (Figure 5d,III), which progressively close into nanotubes (Figure 5d,IV). The similarity observed between the pure ACD fibril cross-section (Figure 5a) and the ACD/CTAB tubule wall thickness (Figure 5c) supports the proposed model. The model is consistent with the reasonable assumption that without CTAB, bile salt derivative alone is unable to form ribbons, as it would cost too much in terms of electrostatic repulsion among the carboxylate heads. It arranges instead in helical fibrils, which allow the best compromise organization for accomplishing intermolecular forces (hydrophobic interactions, hydrogen bonds, and π–π stacking) while keeping the charge heads apart and in contact with water. Upon addition of CTAB, mixed aggregates are formed where the electrostatic repulsion is reduced. This allows the ACD/CTAB tapes to generate without an excessive energy cost and to growth in width thanks to its lower surface charge density, while preserving the bile salt derivative bilayer thickness. The mechanism of transition from ribbons to nanotubes is very similar to what is observed for many other classes of supramolecular filamentous aggregates such as those formed by peptides^61–65^ and nematic elastomer tapes.^66,67^ Initially, twisted ribbons form. However, with a progressively increasing width, a critical width-to-thickness ratio is reached, bending energy becomes more favorable over torsion energy, leading to a switch from twisted to helical ribbons.^64^ Closure of helical ribbons into nanotubes, is finally achieved allowing to reduce the residual line tension associated with the edges of the helical ribbons.^64^

### Nanoparticle Loading

The far-off neutrality stoichiometry provides a very negative charge to the catanionic nanotubes, as demonstrated by a significantly large electrophoretic mobility of about −5.7 μm·cm·V^–1^·s^–1^ for the nanotubes at *X*_ACD_ = 0.9.

This relevant electrophoretic mobility and the very unbalanced surfactant ratio suggest that the nanotubes have a remarkable charge that can be exploited for loading materials through electrostatic interactions. We demonstrated this ability in the loading of positively charged carbon dots and gold nanorods. Cryo-TEM images showed that carbon dots added to the *X*_ACD_ = 0.9 sample fully covered some of the nanotubes (Figure 6a). Some images also show that the interaction with the carbon dots determines the breaking of the nanotubes (Figure 6b). We foresee that the positively charged carbon particles remove ACD molecules from the nanotubes upon direct adsorption on the surface, thereby promoting their destruction. These carbon particles become neutral because of the adsorption of ACD molecules and self-assemble into large amorphous clusters, often imaged on micrographs (Figure S16). In many cases, these clusters decorate the nanotube surface in a second-order interaction (Figure 6c,d) according to a nanotube driven unidirectional assembly. The clusters can be easily separated upon centrifugation and redispersed by heating the sample. Interestingly, full dispersion is accomplished when the temperature approaches the value at which complete temperature-induced nanotube destruction occurs. Separation and redispersion were demonstrated by measurements of the absorbance in the visible region of the sample, before and after centrifugation, and after heating of the centrifuged sample (Figure S17).

**Figure 6:**
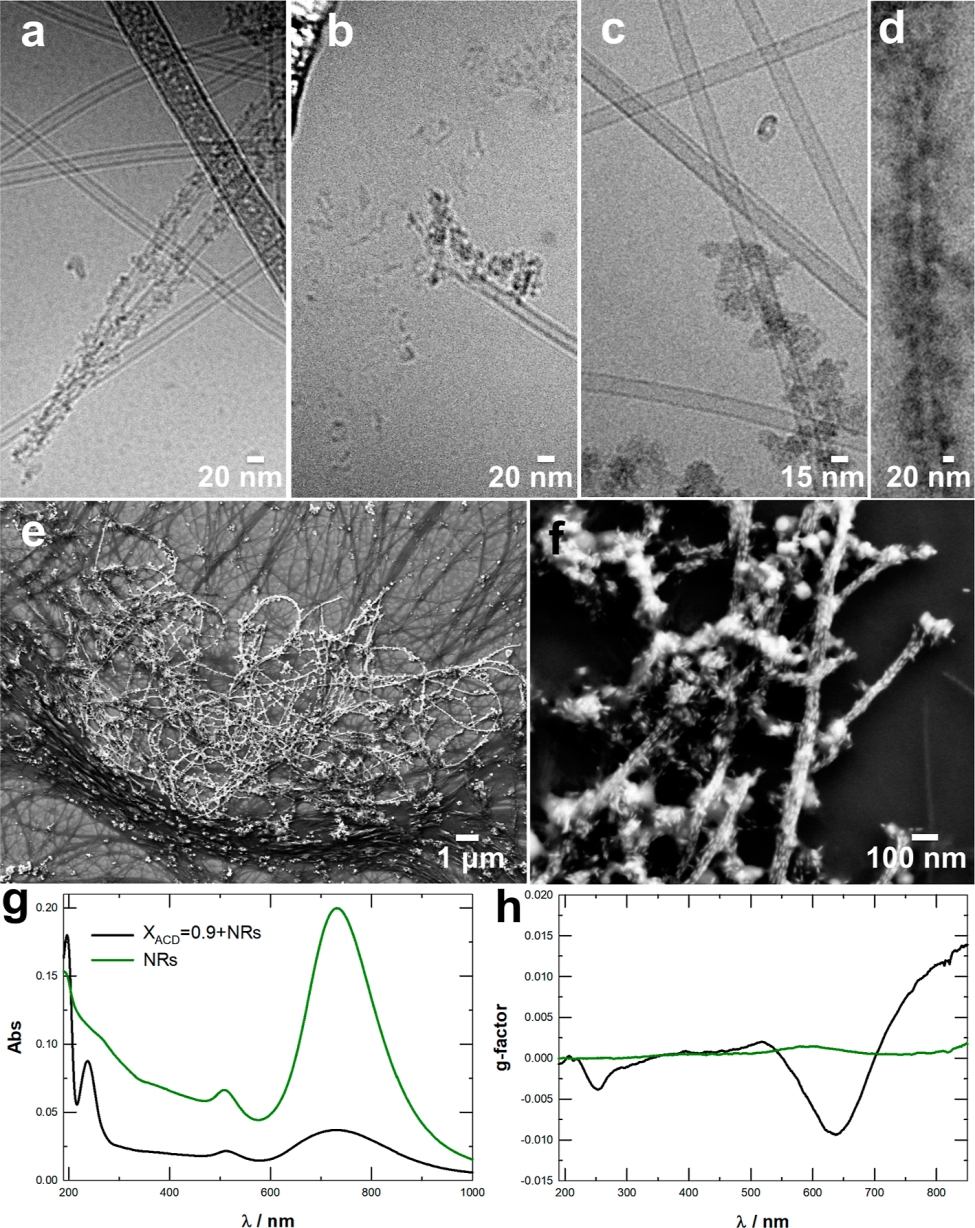
(a–c) Cryo-TEM and (d) TEM image of the negatively charged catanionic tubules interacting with carbon dots in aqueous solution. (e,f) SEM images of gold nanorods interacting with catanionic tubules in aqueous solution. (g) UV–vis spectrum of a 3:1 (v/v) mixture of a catanionic system (*X*_ACD_ = 0.9), mixed with a gold nanorod solution (black line), and UV–Vis spectrum of gold nanorods alone (dark green line). (h) *g*-factors of the same samples.

SEM images also showed a very strong interaction of the nanotubes with positively charged gold nanorods (Figure S18, Figure 6e,f). The interaction dictated remarkable loading and alignment of the nanoparticles on the tubular structures, which also resulted in chiroptical activity in the plasmonic spectral window (Figure 6g,h). As a matter of fact, a CD signal was revealed in this region that is consistent with those generally induced by direct interaction of the nanorods with chiral molecules, like ACD,^70^ or by chiral assembly of nanorods,^71^ which could both contribute to the observed chiroptical response. Notably, the intrinsic chiroptical features of the nanotubes remained preserved in the UV region, thereby demonstrating that the interaction with the nanorods did not compromise the structural chirality of the nanotubes.

## Conclusions

We describe here a catanionic mixture providing self-assembly at a well-defined, very unbalanced charge stoichiometry. The association results in the formation of supramolecular nanotubules at the specific composition of 9 bile acid derivative anions per cetyltrimethylammonium cation, thereby providing catanionic aggregates with a composition much far from the equimolar one. This unconventional self-assembly is reasonably allowed because of a specific very ordered organization of the aggregates at the molecular level, as demonstrated by their intense chiroptical response as well as the well-defined and monodisperse tubular shape. Although it is not trivial to detail the molecular packing, it is clear from the CD that it implies a strong interaction between the ACD molecules with a relevant contribution of the stacked aromatic residues. These conditions can be easily accomplished in the aggregates, considering the much higher fraction of ACD molecules. Concurrently, the intercalation of CTAB molecules is hypothesized. We envision that a bilayer forms the nanotube walls. In such a bilayer, the aromatic residues of the ACD molecules and the hydrophobic chain of CTAB are in the inner part, whereas the charged carboxylic and ammonium heads are located on the surface in contact with the aqueous medium. The well-defined stoichiometry strongly suggests that CTAB is deeply involved in the supramolecular packing, providing a crucial contribution to the stability and curvature of the supramolecular nanotube walls. The association occurs in an alkaline buffer and is promoted by its concentration. The fundamental effect of the buffer indicates that deprotonation of the carboxylic groups of ACD is needed for the CTAB molecule to intercalate and for the supramolecular organization to form. The ammonium head of CTAB contributes to reducing the repulsion among the carboxylic groups on the surface and to strengthening the intermolecular interactions. As the concentration of the buffer is also fundamental, we envision that the buffer is also important to provide a sufficient electrolyte concentration to screen the electrostatic interactions and allow the charged head to pack on the surface of the walls. We have further demonstrated that the nanotubes bind positively charged carbon dots and allow their separation by centrifugation and redispersion upon a temperature increase. The resulting system allows a temperature-controlled carbon dot aggregation, which is a remarkable achievement in light of the wide range of applications based on aggregation-sensitive carbon dot features, such as tunable luminescence,^72,73^ and the control of properties in carbon dots containing composite materials. A substantial loading of positively charged gold nanorods was also reported, leading in turn to an intense chiroptical activity over the plasmonic wavelength range. Tubular architectures draw particular interest for their broad applications in nanotechnology, including catalysis, sensors, and tissue engineering. The ability to control the tubular charge is critical for regulating fundamental functions, such as a carrier, as it dictates electrostatic interactions that are essential for the capacity of the tubule to load molecules (like drugs, DNA, or proteins) or nanoparticles as well as its ability to aggregate and penetrate biological membranes. We demonstrate here that mixtures of oppositely charged surfactants can be exploited to fabricate highly charged tubules at very low concentrations, where the presence of free monomers is very low and the interactions with other materials can be optimized. These outcomes could be of very general interest in material science and promote the investigation of rationally designed mixtures of oppositely charged surfactants as an important tool for the preparation of nanostructures for a large set of applications.

## Supplementary Material



## Notes

FTIR and NMR data; DLS, SAXS, CD, and UV data; SAXS fitting parameters; AFM images and profiles; and Cryo-TEM images and statistics (PDF)
