# Supplementary material for: Exact Stoichiometry Far-Off Neutrality Catanionic Nanotubes: Unconventional Self-Assembly of Oppositely Charged Surfactants
Source: J Am Chem Soc. 2026 Apr 30;148(18):18733–45. doi: 10.1021/jacs.5c21811 (PMC13185106; doi:10.1021/jacs.5c21811)
Supplement: Supplementary file 1 [file ja5c21811_si_001.pdf]

Exact stoichiometry far-off neutrality catanionic nanotubes: unconventional self-assembly of oppositely charged surfactants

Valerio La Gambina[a], Lorenzo A. Rocchi[a], Simona Sennato[bc], Milad Radiom[d], Karin Schillén[f], Crispin Hetherington[e], Iolanda Francolini[a], Fabrizio Vetica[a], Francesca Leonelli[a], Alessandra Del Giudice[a*], Maria Chiara di Gregorio[a*], Raffaele Mezzenga[dg], Luciano Galantini[a*]

a Department of Chemistry, Sapienza University of Rome, Rome, 00185, Italy;

b Department of Physics, Sapienza University of Rome, Rome, 00185, Italy;

c National Research Council–Institute for Complex Systems (CNR-ISC), Rome, 00185, Italy;

d Department of Health Sciences and Technology, ETH Zürich, Zürich, 8092, Switzerland;

e National Center for High Resolution Electron Microscopy, Centre for Analysis and Synthesis, Lund University, Lund, SE-22100, Sweden;

f Division of Physical Chemistry, Department of Chemistry, Lund University, Lund, SE-22100, Sweden;

g Department of Materials, ETH Zürich, Zürich, 8093, Switzerland;

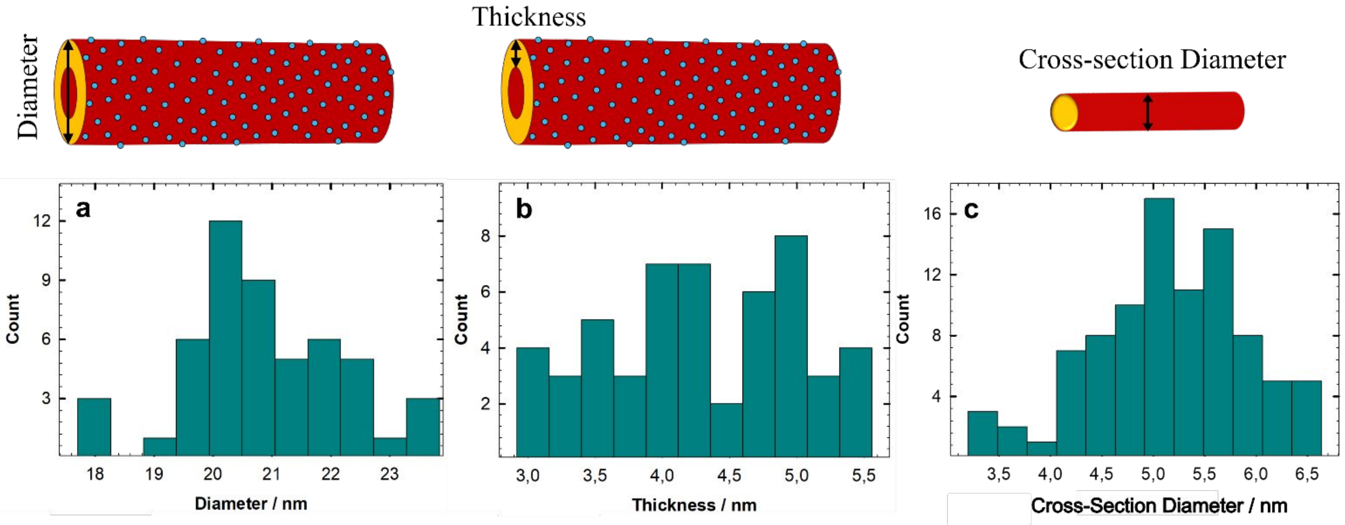

Figure S1. Size statistics based on cryo-TEM images related to a) tubule diameter, b) tubule wall thickness and c) fiber cross-section diameter.

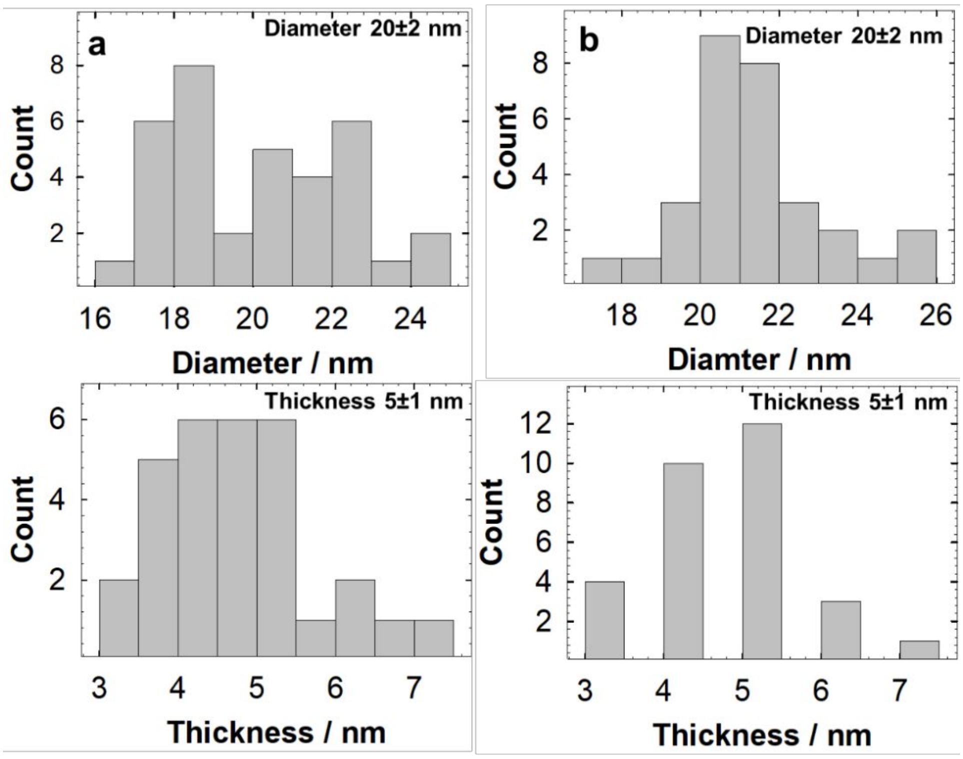

Figure S2. Size statistics based on cryo-TEM images of mixtures related to tubule diameter and wall thickness at a) X_{ACD} = 0.975 and b) X_{ACD} =0.8.

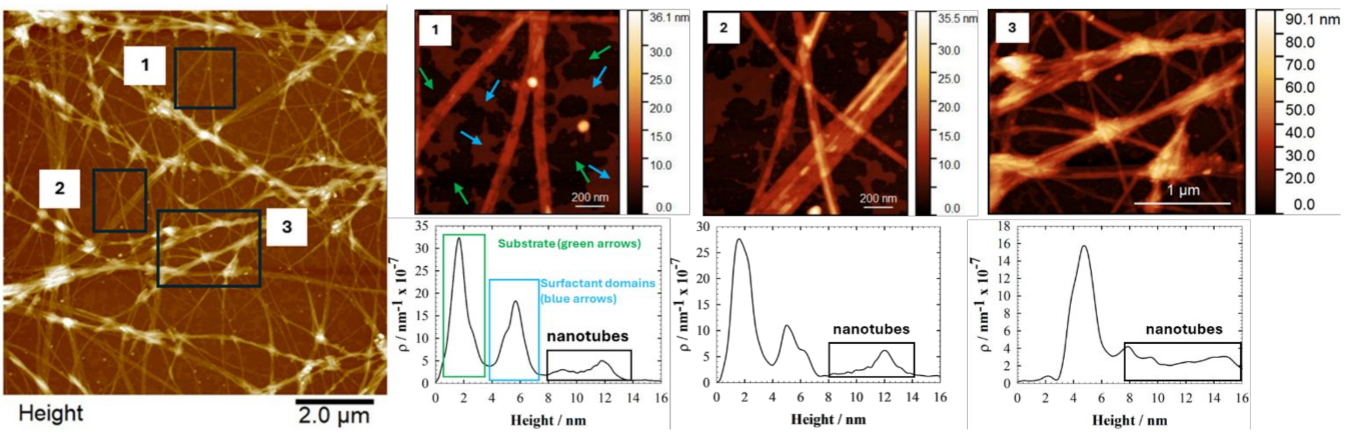

Figure S3. Height analysis of the mixture with X_{ACD} = 0.9 and total concentration 1 mM based on AFM images.

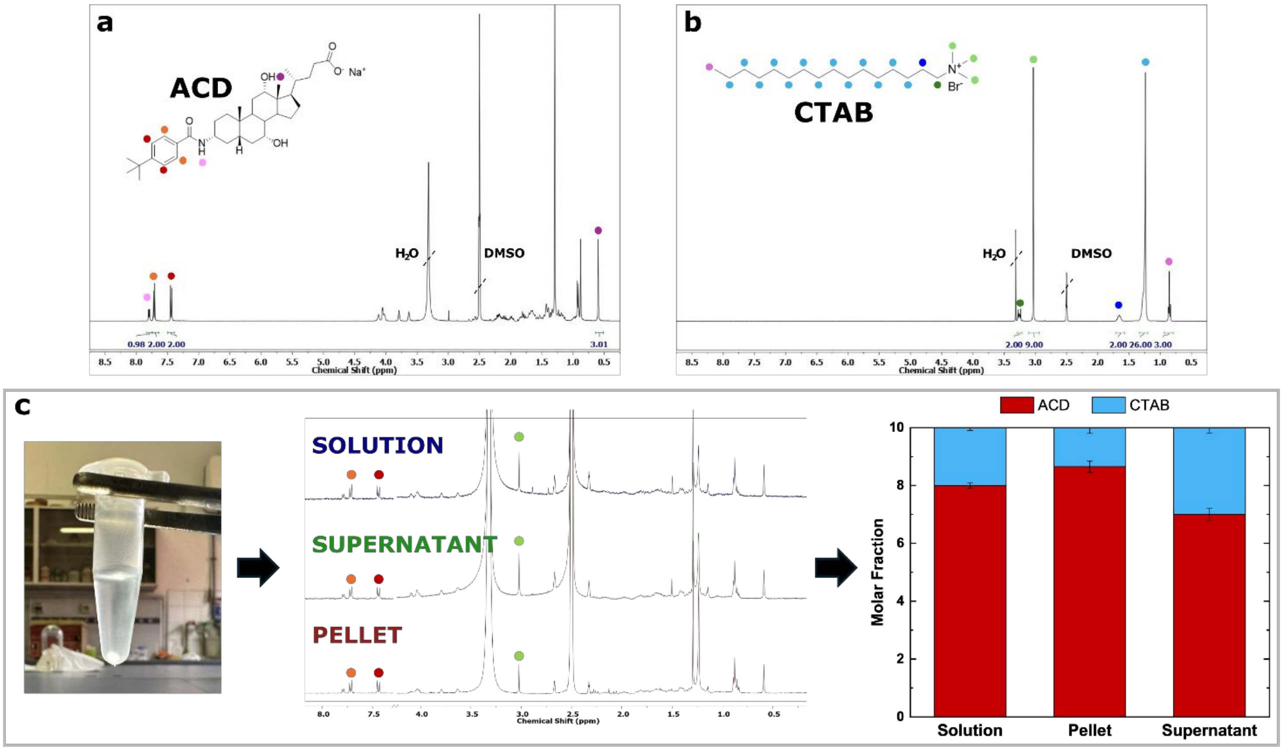

Figure S4. ^{1}H-NMR spectra of a) pure ACD, b) pure CTAB, and c) the catanionic mixture with X_{ACD} =0.8 in 30 mM carbonate buffer. All NMR spectra were recorded in deuterated DMSO. The catanionic mixture was analyzed both as prepared and after centrifugation at 14800 rpm, for 1 h. Centrifugation yielded a pellet (precipitated fraction) and a supernatant, which were measured separately.

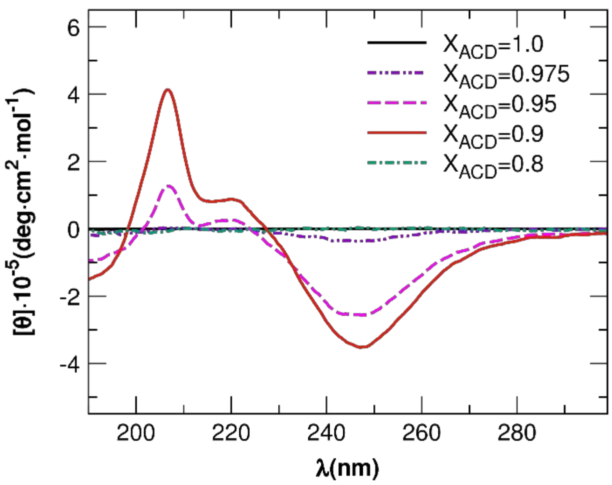

Figure S5. CD curves of the pure ACD solution and the ACD/CTAB mixtures at different ACD fractions in 30 mM carbonate/bicarbonate buffer. The total surfactant concentration was 0.6 mM.

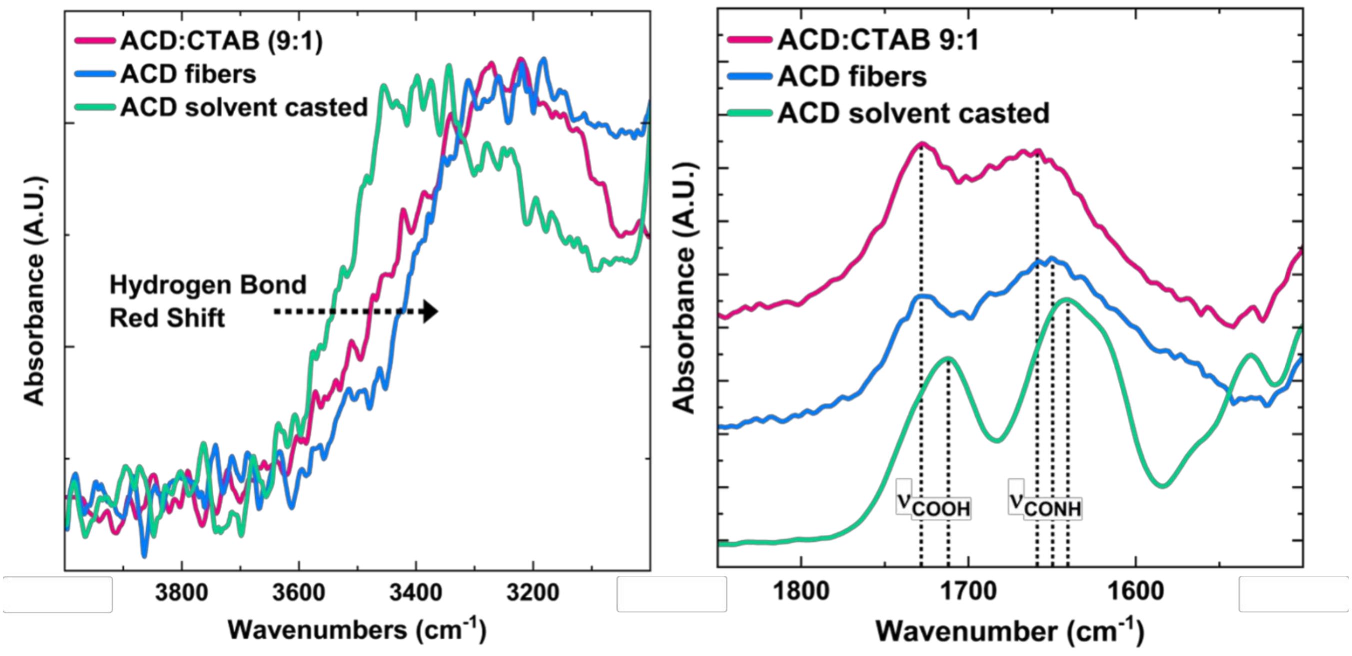

Figure S6. FTIR spectra of the dried samples highlighting the OH and COOH region.

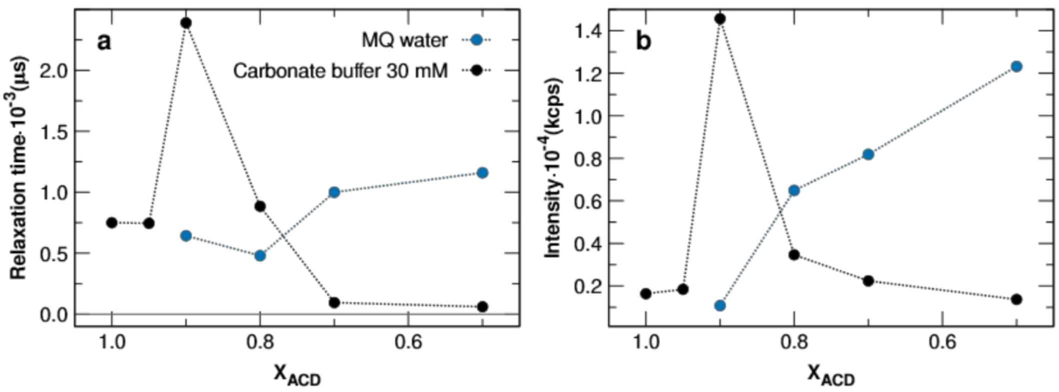

Figure S7. Comparison of catanionic mixtures aggregation in pure water and in 30 mM $NaHCO_3/Na_2CO_3$ buffer, as detected by dynamic light scattering. a) Relaxation time as a function of X_{ACD} b) Derived count rate as a function of X_{ACD}.

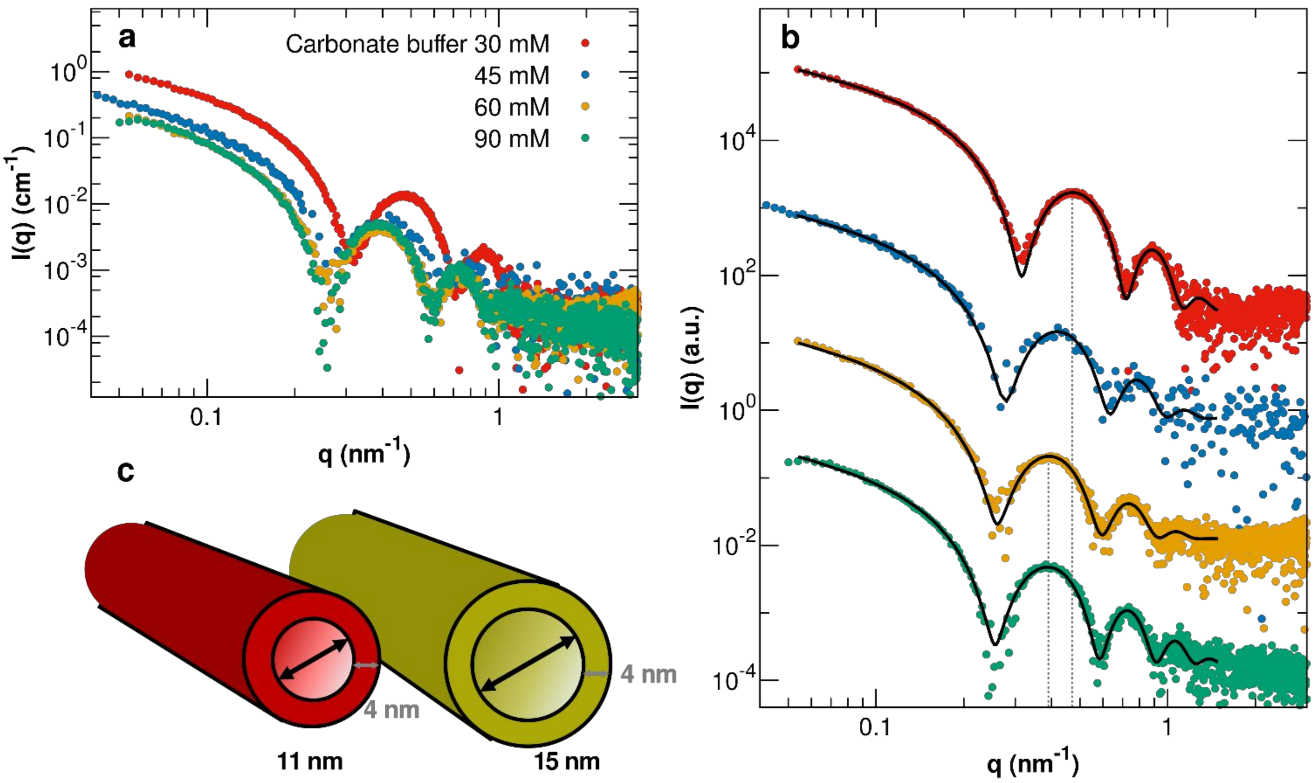

Figure S8. SAXS curves of the X_{ACD} = 0.9 mixture at different carbonate/bicarbonate buffer concentrations reported as a) macroscopic scattering cross sections on absolute scale. b) SAXS data of panel a) are shown scaled for improved visualization, together with model form factors of a long hollow cylinder. Best fitting parameters are reported in Table S4.

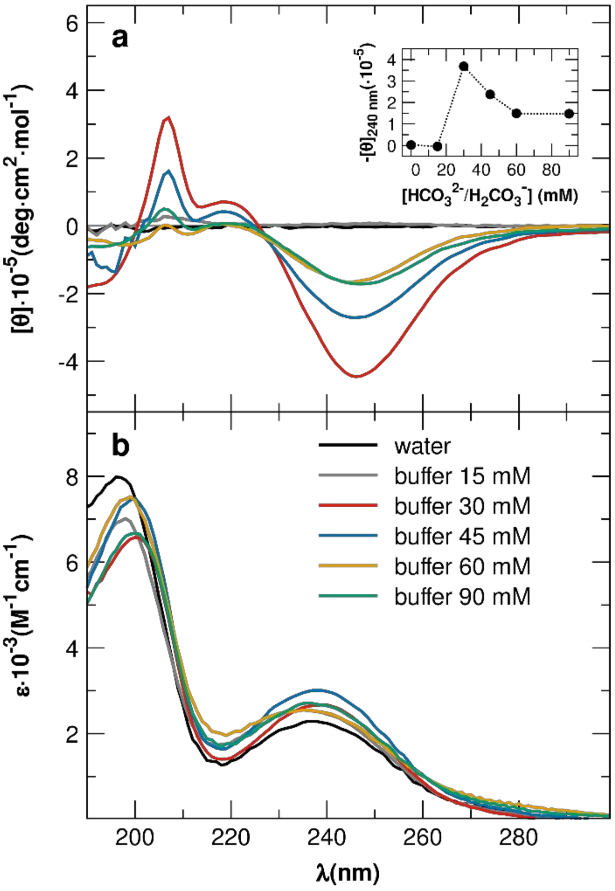

Figure S9. a) CD curves of the X_{ACD} = 0.9 mixture at different carbonate/bicarbonate buffer concentrations, and b) corresponding UV absorption curves.

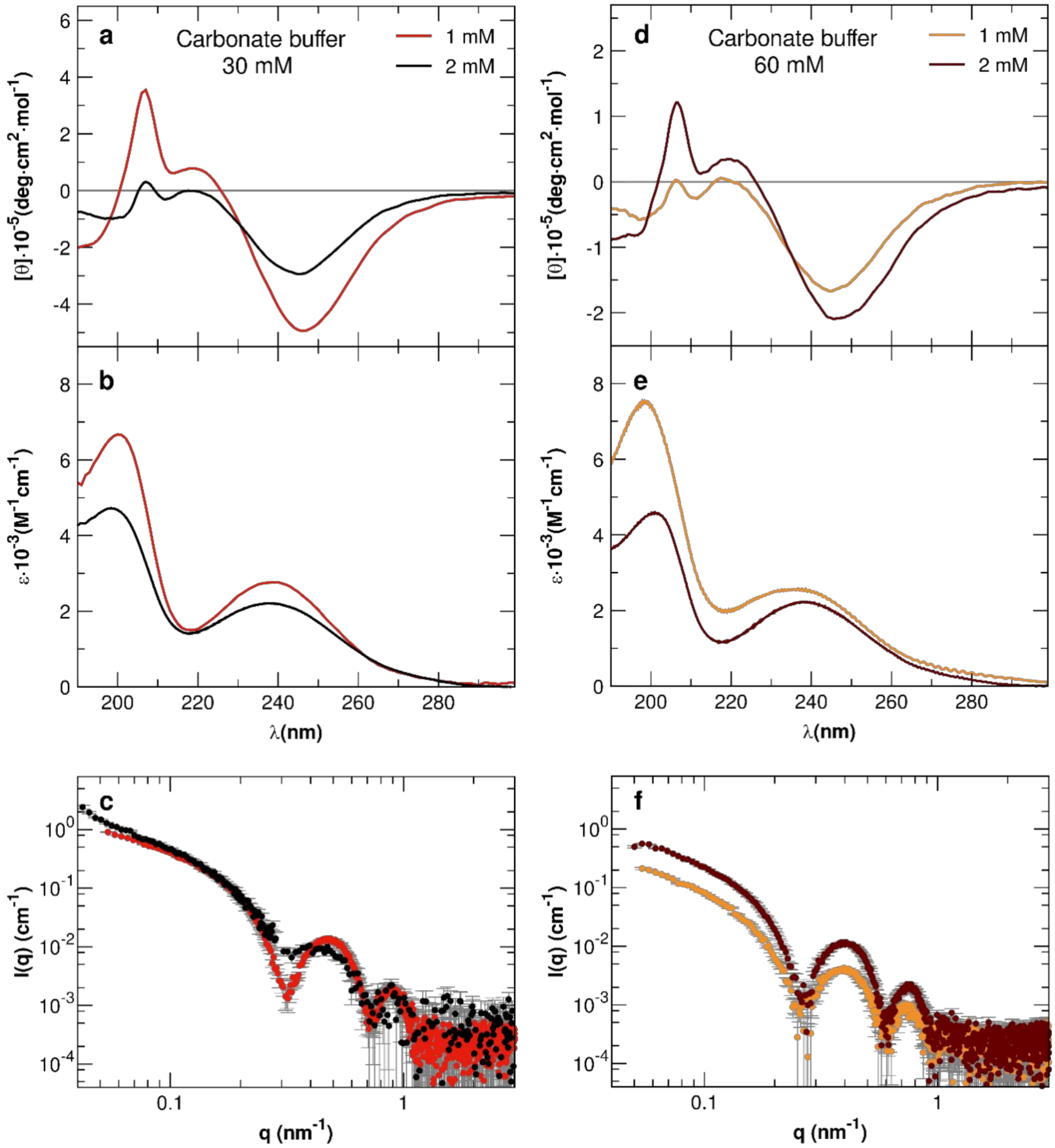

Figure S10. CD, UV and SAXS curves of the X_{ACD} = 0.9 mixture at 1 and 2 mM total surfactant concentration in carbonate buffer of concentration a,b,c) 30 mM and d,e,f) 60 mM.

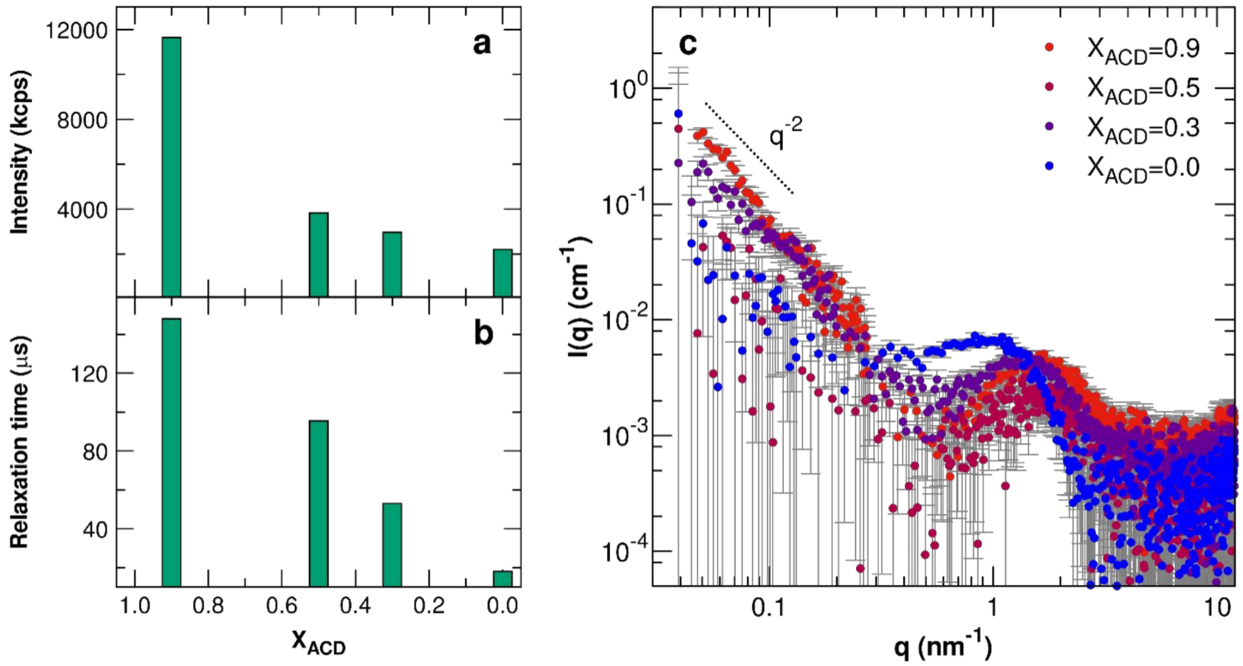

Figure S11. Dynamic light scattering a) average scattering intensities and b) relaxation time as a function of the ACD surfactant molar fraction X_{ACD} for mixtures at total surfactant concentration of 10 mM. c) SAXS curves of 10 mM CTAB and ACD/CTAB mixtures in 30 mM carbonate/bicarbonate buffer at different ACD fractions reported as macroscopic scattering cross sections on absolute scale. The SAXS profile of a pure CTAB solution (10 mM) shows a characteristic oscillation due to the surfactant micelles, that moves to larger q in the presence of ACD, with the highest low-q intensity seen for X_{ACD} =0.9. However, the characteristic oscillations from the hollow cylinder form factor are not observed, and the low-q intensity is of the same order of magnitude as for the mixtures at 1 mM despite the total surfactant concentration is ten times higher in this case.

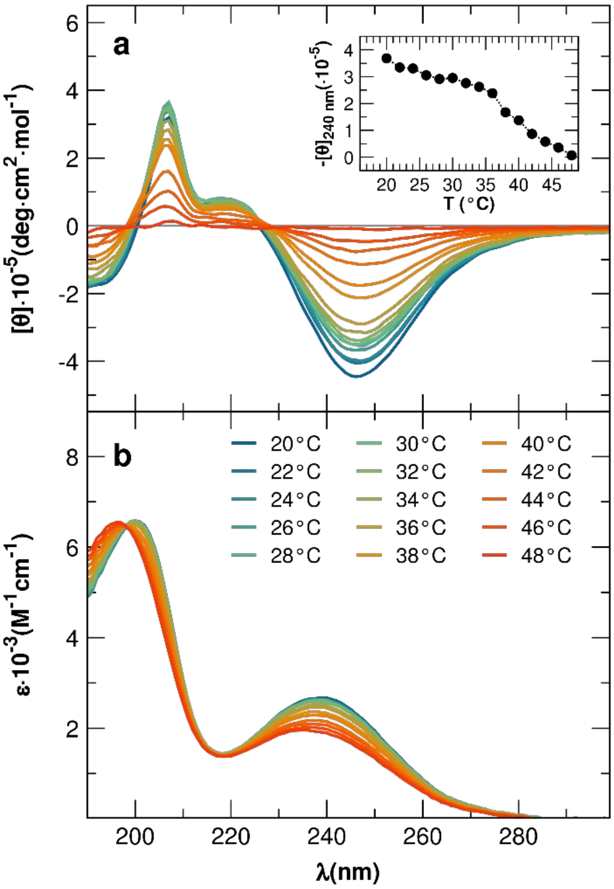

Figure S12. CD curves of a) the X_{ACD} = 0.9 mixture at different temperatures (in the range 20-48 °C), and b) corresponding UV absorption curves. In the inset in panel a) the CD signal at 240 nm is shown as a function of temperature.

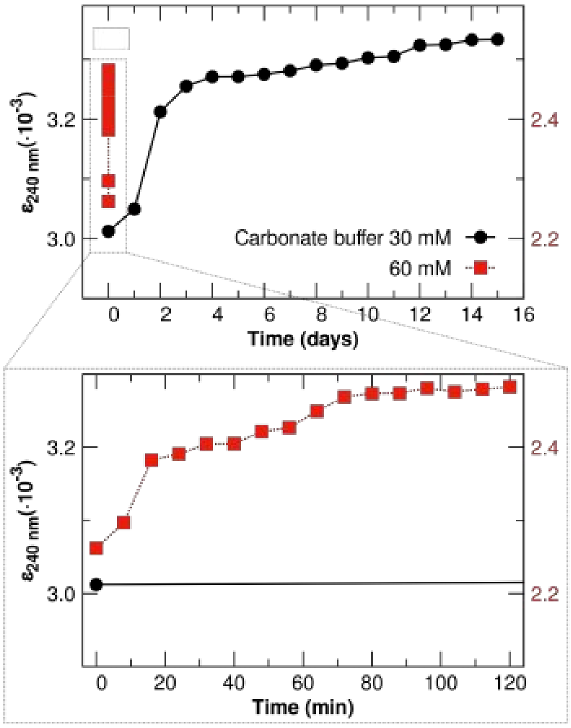

Figure S13. kinetics of formation of catanionic tubules followed through UV spectroscopy at 30 and 60 mM buffer concentrations.

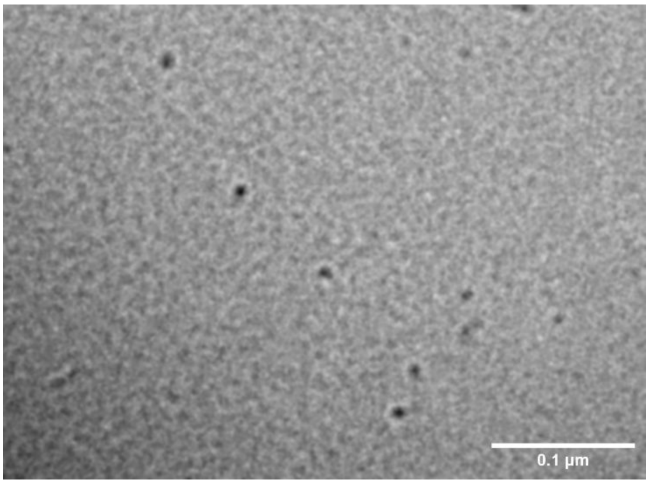

Figure S14. Cryo-TEM image of the X_{ACD} = 0.9 mixture in buffer 30 mM at time 0 from preparation.

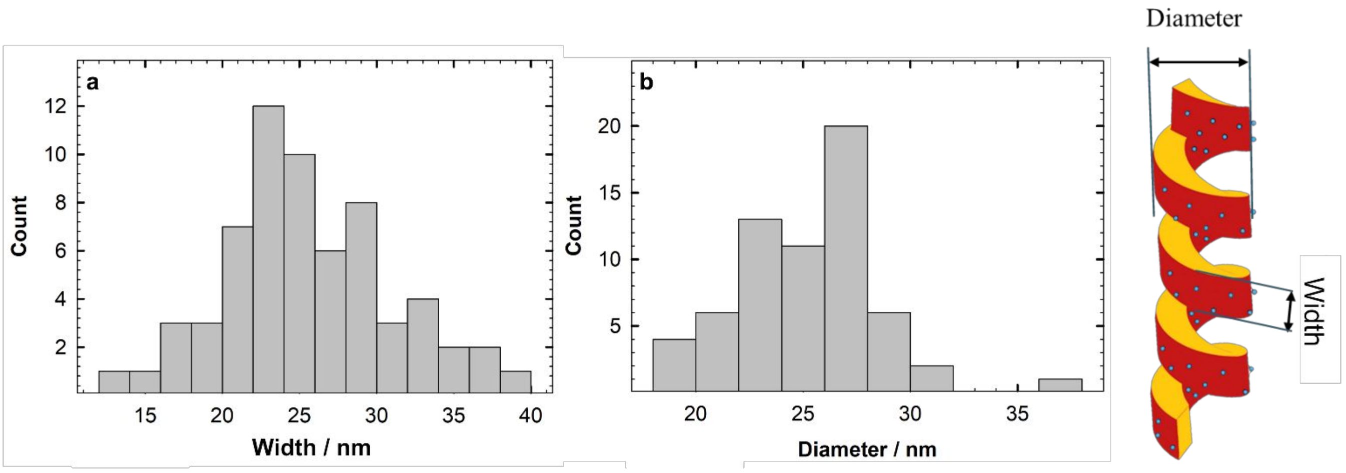

Figure S15. Size statistics based on cryo-TEM images related to a) ribbon width and b) ribbon diameter.

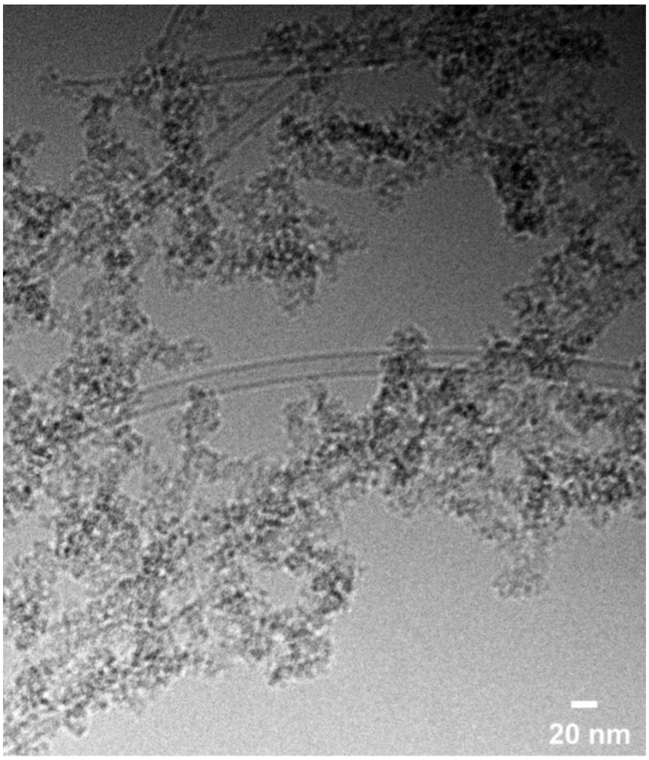

Figure S16. Cryo-TEM of the ACD/CTAB mixture at X_{ACD} = 0.9 interacting with a water dispersion of carbon dots (1:3 in volume) that shows the presence of clusters of carbon dots formed upon interactions with ACD molecules.

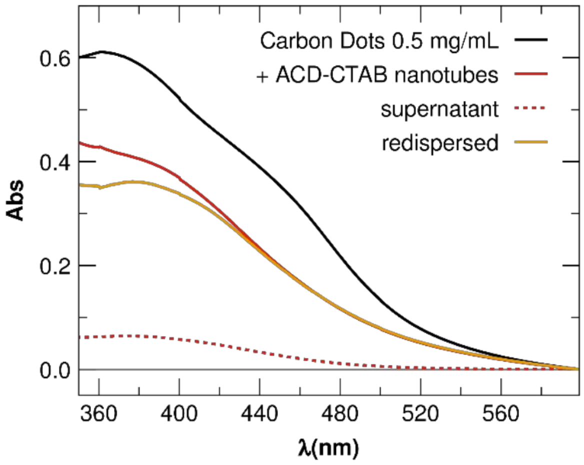

Figure S17. UV absorption spectra of 0.5 mg/mL water dispersion of carbon dots (black line), mixture of the same water dispersion 3:1 in volume with the X_{ACD} = 0.9 mixture pre-centrifuge (red line), the supernatant after centrifugation (red-dotted line) and same mixture after redispersing the carbon dots in solution by breaking the tubules with temperature (dark-yellow line).

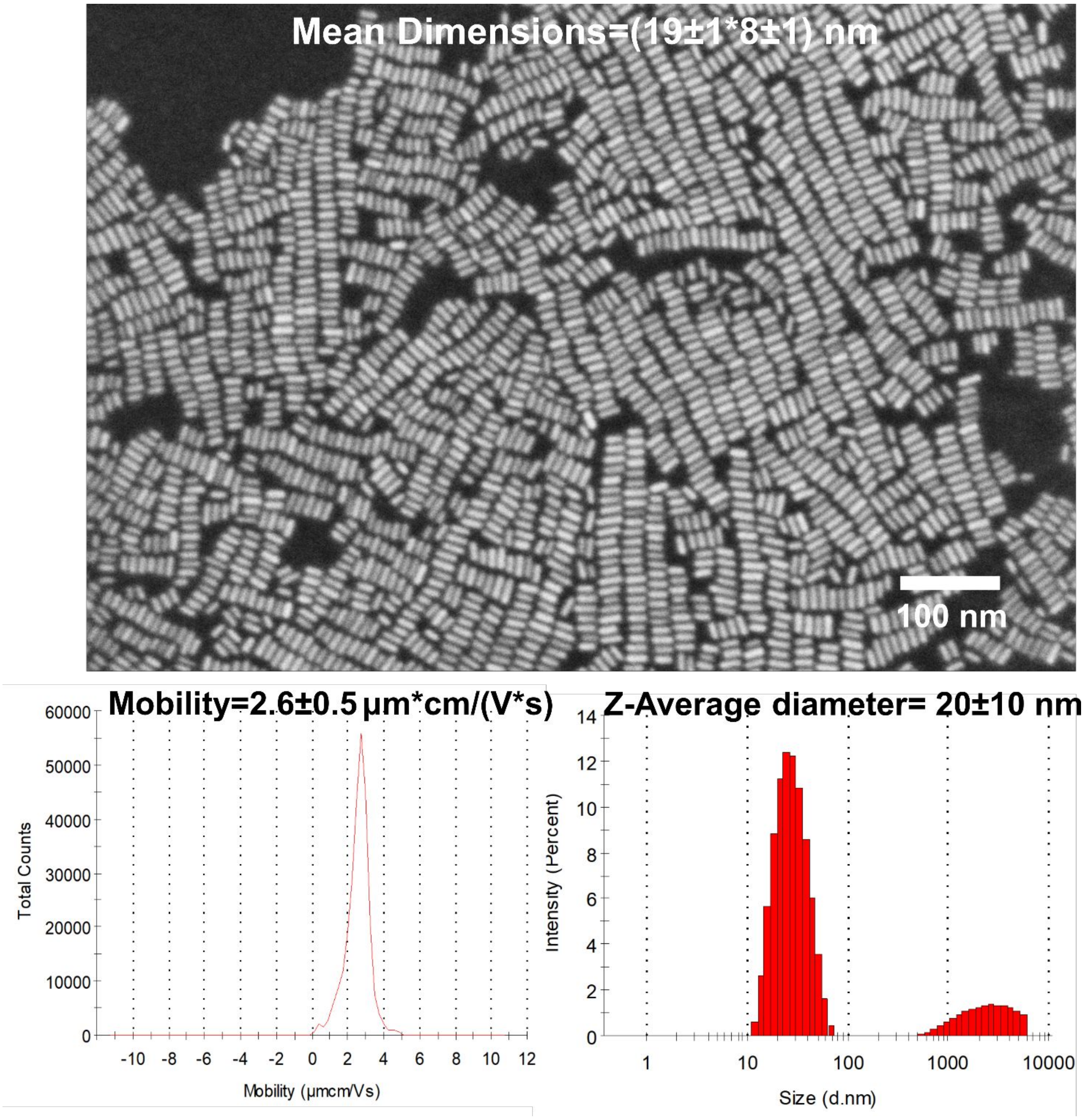

Figure S18. SEM, DLS size distribution and electrophoretic mobility measurement of the gold nanorods ([Au^0] = 0.5 mM) used for the interaction with the nanotubes.

Table S1. SAXS best fitting parameters of Figure 3b.

Model	Hollow Cylinder
X_{ACD}	0.9
Volume fraction	$(4.33\pm0.03)\cdot10^{-4}$
Background (cm^{-1})	$(2.3\pm0.2)\cdot10^{-5}$
Radius (nm)	5.63±0.02
Thickness (nm)	3.85 ±0.03
Length (nm)	200
SLD (10^{-6} Å^{-2})	10.88
SLD solvent (10^{-6} Å^{-2})	9.4
Thickness width	0.1
χ^2	1.19

SAXS best fitting parameters of X_{ACD} = 0.9 in 30 mM carbonate/bicarbonate buffer of Figure 3 b. Grey parameters are fixed during the optimization. The scattering length density (SLD) value for the hollow cylinder wall was calculated assuming the X_{ACD} = 0.9 composition and molar volumes of 495 cm^3/mol for ACD and 338 cm^3/mol for CTAB, and a number of electrons consistent with the empirical formulas $C_{35}H_{52}O_5NNa$ and $C_{16}H_{33}N(CH_3)_3Br$, respectively. These values, together with the known concentration, imply a volume fraction of $4.79\cdot10^{-4}$, not far from the best-fit value for the hollow cylinder model. The length of the aggregates is above the detectable limit imposed by the minimum q value accessible (> 70 nm) and was fixed at the arbitrary value of 200 nm in the modeling.

Table S2. SAXS best fitting parameters of mixtures showing nanotube SAXS features in Figure 3c.

Model	Hollow Cylinder				
X_{ACD}	0.975	0.95	0.9	0.8	0.7
Volume fraction	$(1.62\pm0.02)\cdot10^{-4}$	$(1.78\pm0.02)\cdot10^{-4}$	$4.79\cdot10^{-4}$	$(1.64\pm0.02)\cdot10^{-4}$	$(0.42\pm0.02)\cdot10^{-4}$
Background (cm^{-1})	$3\cdot10^{-4}$	$3\cdot10^{-4}$	$3\cdot10^{-4}$	$3\cdot10^{-4}$	$3\cdot10^{-4}$
Internal radius (nm)	6.18	6.18	6.18±0.02	6.18	6.18
Wall thickness (nm)	4.10	4.10	4.10±0.01	4.10	4.10
Length (nm)	200	200	200	200	200
SLD (10^{-6} Å^{-2})	10.88	10.88	10.88	10.88	10.88
SLD solvent (10^{-6} Å^{-2})	9.4	9.4	9.4	9.4	9.4
Thickness width	0.1	0.1	0.1	0.1	0.1
χ^2	1.66	2.40	3.36	2.98	1.80

SAXS best fitting parameters of the ACD/CTAB mixtures in 30 mM carbonate/bicarbonate buffer at different ACD fractions of Figure 3c according to the hollow cylinder model. Grey parameters are fixed during the optimization.

Table S3. SAXS best fitting parameters for Figure 3c.

Model	Sphere (A)+ Flexible Cylinder (B)
X_{ACD}	1
Background (cm^{-1})	(1.9±0.2)·10^{-4}
SLD (10^{-6} Å^{-2})	11
SLD solvent (10^{-6} Å^{-2})	9.4
A volume fraction	(1.08±0.07)·10^{-4}
A radius (nm)	2.83±0.07
B volume fraction	(0.16±0.01)·10^{-4}
B radius minor (nm)	2.83
B Kuhn length (nm)	15
B length (nm)	10000
χ^2	1.09

SAXS best fitting parameters of the pure ACD 1 mM in 30 mM carbonate/bicarbonate buffer of Figure 3c, not described by the hollow cylinder form factor suitable for the X_{ACD} = 0.9 mixture. Grey parameters are fixed during the optimization. The SLD value was calculated assuming a molar volume of 495 cm^3/mol for ACD. The fibril length modeled as a flexible cylinder, was fixed at the arbitrary value of 10000 nm, being outside of the SAXS detectable range.

Table S4. SAXS best fitting parameters of Figure S8

Model	Hollow Cylinder			
X_{ACD}	0.9			
$NaHCO_3/Na_2CO_3$ buffer (mM)	30	45	60	90
scale	(433±3)x10^{-6}	(133±1)x10^{-6}	(754±9)x10^{-7}	(96±2)x10^{-6}
background	(23±2)x10^{-5}	(30±4)x10^{-5}	(248±7)x10^{-6}	(17±1)x10^{-5}
Radius (nm)	5.63±0.02	6.67±0.04	7.03±0.04	7.69±0.04
Thickness (nm)	3.85±0.03	3.85	4.25±0.06	3.37±0.07
Length (nm)	200	200	200	200
SLD (10^{-6} / Å^2)	10.88	10.88	10.88	10.88
SLD solvent (10^{-6} / Å^2)	9.4	9.4	9.4	9.4
Thickness width	0.1	0.1	0.1	0.1
χ2	1.1939	1.1188	1.2958	1.2871

Table S4. SAXS best fitting parameters of mixtures at X_{ACD} = 0.9 in different concentrations of carbonate/bicarbonate buffer. Grey parameters are fixed during the optimization.
